# A systematic literature review of the stereotype content model in the fields of psychology and marketing: main themes examined in the literature and an agenda for future research in marketing

**DOI:** 10.3389/fpsyg.2024.1392629

**Published:** 2024-05-20

**Authors:** Gonzalo Luna Cortes

**Affiliations:** Jönköping International Business School, Jönköping University, Gjuterigatan, Jönköping, Sweden

**Keywords:** stereotype content model, warmth, competence, agency, communion, systematic literature review

## Abstract

The stereotypes content model indicates that two traits (i.e., warmth and competence) govern individuals’ impression formation. The great variety of research that has used this theory since the early 2000s leads to a need for a structured overview of prior findings. The goal of this study is to provide a concise map of research streams and present a research agenda. We conducted a systematic literature review of 955 articles. A bibliographic coupling analysis showed four clusters, i.e., (1) the general theoretical foundations of the SCM, (2) the societal impact of key stereotypes (with emphasis on gender), (3) research in clinical psychology and child development, and (4) marketing. Taking a closer look at research in marketing (using co-occurrence analysis), six research streams were identified, including research on branding, country-of-origin, front-line service providers, prosocial consumer behavior, perception of endorsers, and, more recently, on artificial intelligence (AI). The review presents key findings and research gaps across these topics. Finally, we reviewed the few articles that, although not falling into these streams, opened important research veins. This process provided the essential information to present a promising and complete research agenda, to continue building knowledge with impactful implications in different contexts.

## 1 Introduction

The stereotype content model (SCM) (Fiske et al., 2002) is the most widely used theory for research on stereotypes (Halkias and Diamantopoulos, 2020). The theory stipulates that two fundamental dimensions guide individuals’ impression formation, namely, “warmth and competence.” Warmth is associated with friendliness and kindness, while competence is associated with efficacy and skill (Fiske et al., 2007). The SCM predicts distinct affective and behavioral responses by individuals toward other people and/or social groups (Fiske et al., 2007; Cuddy et al., 2008; Halkias and Diamantopoulos, 2020).

The theory—and terminology—was coined by Fiske et al. (1999, 2002), who considered some early interpretations of social judgment as the basis for their model (Asch, 1946; Zanna and Hamilton, 1972). Since then, these two dimensions have been applied to examine how individuals evaluate others, and specific groups of people (Cuddy et al., 2007). In the fields of management and marketing, some authors have examined the effect of these two dimensions on managers’ perception of employees (Cuddy et al., 2011), consumers’ perception of companies (Aaker et al., 2010), brands (Aaker et al., 2012; Xue et al., 2020), and service providers (Güntürkün et al., 2020). Most current research topics include the perception of AI and robot service providers (e.g., Meyer and Asbrock, 2018; Grazzini et al., 2023), the content shared on social media and the perception of influencers (Ren et al., 2023), perceived language used to address consumers formally (vs. informally) (Leung et al., 2023), vegan stereotypes (Adamczyk and Maison, 2023), and the effect of stimuli on sustainable tourists’ behaviors (Chua et al., 2023).

While there is a wide variety of topics examined in the literature, there are two main fields where most prior studies have been conducted, namely, in psychology (e.g., Imhoff et al., 2013; Vervecken et al., 2015; Meyer and Asbrock, 2018; Grigoryev et al., 2019; Xue et al., 2020; Brender-Ilan and Reizer, 2021; Gärtner et al., 2022; Linne et al., 2022) and marketing (e.g., Kolbl et al., 2019, 2020; Diamantopoulos et al., 2021; Gidaković et al., 2022). In fact, prior research in marketing on the SCM uses the main theoretical basis of research in social psychology, connecting prior research from these two fields. For instance, research on the perception of service providers (Wang et al., 2017; Li et al., 2019) and mental health (Gärtner et al., 2022; Seewald and Rief, 2023) use the same theoretical bases (i.e., Fiske et al., 1999, 2002, 2007; Cuddy et al., 2008), and are associated due to the effect of these two traits on the patients’ (i.e., the clients’) perception and satisfaction with their therapist (i.e., the service provider) (Seewald and Rief, 2023). The present review narrows the scope to these two areas of knowledge. Among these, recent authors have mentioned the emerging interest among scholars in the SCM and its implications for the fields of marketing (Ren et al., 2023) and consumer research (Leung et al., 2023). The recent interest among scholars in marketing and the amount of research conducted (see Gidaković et al., 2022) leads to a need to synthesize prior findings in this field. Following this need for research, in the present study, we first (1) provide an overview of research in psychology subfields, which form the basis of the SCM, and next, (2) we take a closer look at prior findings in marketing.

Given the large number of subfields in psychology and marketing where this theory has been applied, there is a need for a structured overview of research on the SCM. A review can provide a concise map of general themes, and observe current gaps among them. The latter can serve as a guide for upcoming research on warmth and competence (hereafter W/C). This need for research is the motivation for the present study, leading to the following research questions: (1) Which are the main themes in the fields of psychology and marketing that use the SCM framework, and what are the most important prior findings? Following the above-mentioned need for research in the fields of marketing, we also strive to answer the following research question: (2) Which are the most important topics to explore in future research in marketing?

This paper a systematic review on research in psychology and marketing that use the SCM, which can serve as a tool for future theoretical frameworks that use this theory, to select the most appropriate works, and provide new value to the field of marketing. The paper is structured as follows. First, we present the method and data used to conduct the review, including the source and procedure followed, how the articles were identified, and the selection criteria. Next, a summary of descriptive results is presented, including a description of fields of research where the SCM has been applied, journals where most prior work has been published, years of publication, authors, geographic areas, citations, and main theories. In addition, the study presents the main themes examined in the literature based on a bibliographic coupling analysis of the documents used for this review. Building on this analysis, prior findings from the different fields are presented, with emphasis on research in marketing. To provide a concise view of prior topics examined in marketing, we present a co-occurrence analysis focusing on this field. The study ends with a discussion of the results and a description of the most promising veins for future research. We place special emphasis on future research veins in marketing, following the urgent need for research identified by previous authors in the field.

## 2 Method and data

### 2.1 Procedure and source

We performed a systematic literature review on the SCM in *business and management*, and in *psychology*. This method is extensively used in the literature to thoroughly review a research topic with a high number of publications over a relatively long period (e.g., Bai et al., 2019; Koutsimani et al., 2019; López-Valenciano et al., 2021; Luna-Cortés et al., 2022a; Luna-Cortes, 2023, 2024; Luna-Cortes and Brady, 2024). We carried out the data extraction using Scopus. As recommended, we used only one dataset to mitigate data homogenization issues faced when working with multiple databases (Mariani et al., 2022). We used Scopus as it is known to have a larger coverage than WoS (Mariani et al., 2022). We performed the article retrieval from Scopus on August 8th, 2023.

### 2.2 Identification of articles

In the first step, we identified all potential articles that might form part of the dataset for our study. Following prior studies, we selected a series of keywords associated to the SCM, i.e., *stereotype, warmth, warm, competence, competent* (Halkias and Diamantopoulos, 2020)*, agency* and *communion* (Koch et al., 2016). We included the option “OR” (i.e., appearance of any of these words) in the “Title,” “Keywords” *or* in the “Abstract.” We limited the search to fields of “Psychology” and “Business, management, and accounting.” The initial hit was 1,147 documents. By organizing the articles alphabetically by “title,” it could be observed that some of the articles were repeated (i.e., 18 double entries). Repeated articles were removed. After this, we obtained an initial dataset with 1,129 documents. Not all these documents were empirical articles, there were some not in English, etc. Thus, selection criteria were established to choose relevant articles for the review, as indicated next.

### 2.3 Selection criteria

The abstracts of the studies selected were carefully read by the authors, and further inclusion was decided based on the following criteria:

1. *Type of study*: the articles needed to focus on the SCM (i.e., the effect of W/C on impression formation and further outcomes).
2. *Publication status and language*: we included only Journal articles. The articles should be in English and peer reviewed.
3. *Year of publication:* the first studies that settled the basis of the effect of stereotypes on impression formation appeared in the 90s (e.g., Copley and Brownlow, 1995; Fiske et al., 1999). Thus, we selected a range from 1990 to 2023 (studies before 1990 were excluded).
4. *Study design*: the studies should be empirical. Hence, the studies needed to include a method, either qualitative or quantitative, to capture levels of warmth or competence (or synonyms used in research, such as agency to refer to competence) and their effect on variables of interest in psychology or marketing (including consumer behavior).
5. *In the field of business and management—only studies in marketing*: finally, we performed an individual review of articles in the field of business and management, to only gather articles that belonged to marketing, therefore, avoiding unrelated subareas, such as organization management.

Following these criteria, 125 articles were removed because either they did not focus on the stereotype content model (*N* = 99), published before 1990 (*N* = 11), they were not empirical (*N* = 9), or were not in English (*N* = 6). As a result, 1,024 articles were selected at this stage. Finally, through the individual review of research in the field of business and management (to keep only research in marketing), we removed 69 articles, leading to a final dataset with 955 articles.

**Figure 1:**
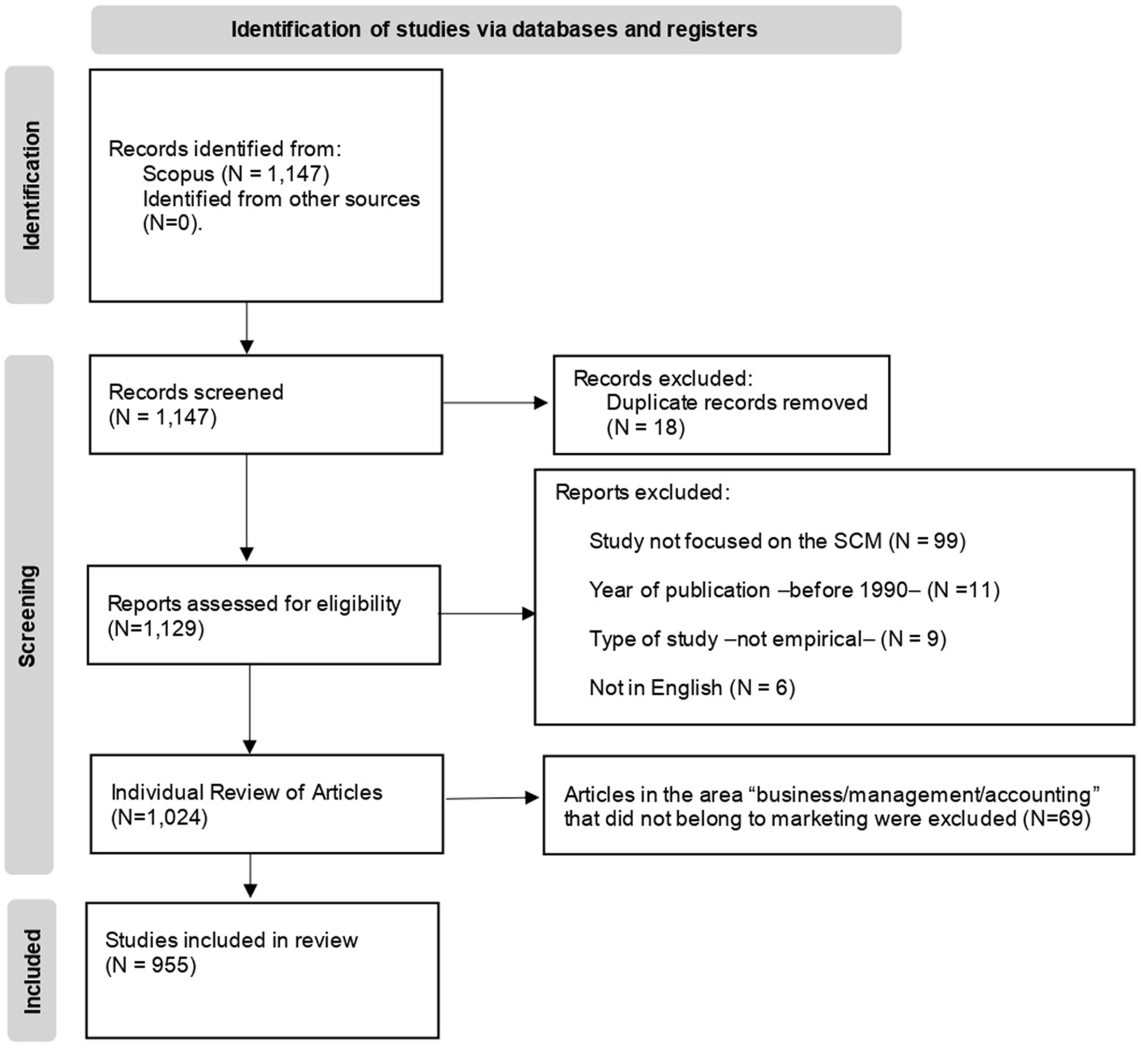
PRISMA diagram describing the process and criteria used to obtain the dataset.

A PRISMA flowchart is presented next in Figure 1, which shows the step-by-step process of the application of inclusion and exclusion criteria to generate a final number of studies for analysis in the systematic review (Page et al., 2021).

## 3 Summary of descriptive results

### 3.1 Fields, year of publication, authors, geographical areas, and citations

**Figure 2:**
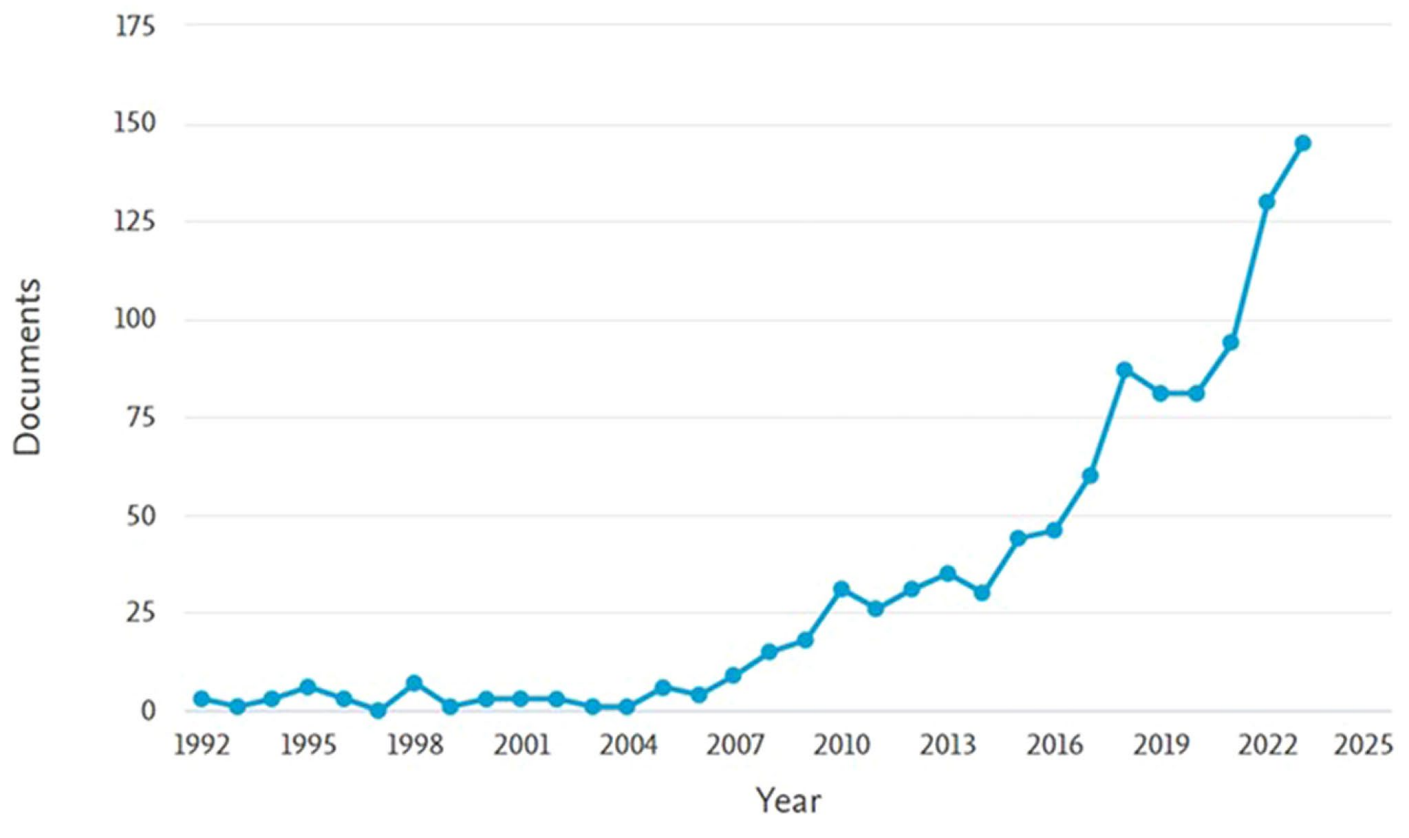
Number of articles published per year.

The descriptive analysis showed that 69% of the studies (*N* = 662) were published in psychology, while 31% (*N* = 293) of research were published in marketing. The year when more articles have been published is 2023 (*N* = 145), followed by 2022 (*N* = 130), and 2021 (*N* = 94). This shows that the majority of research has been published in the last few years, with more than 50% published since 2019 and more than 80% in the last decade (see Figure 2).

**Figure 3:**
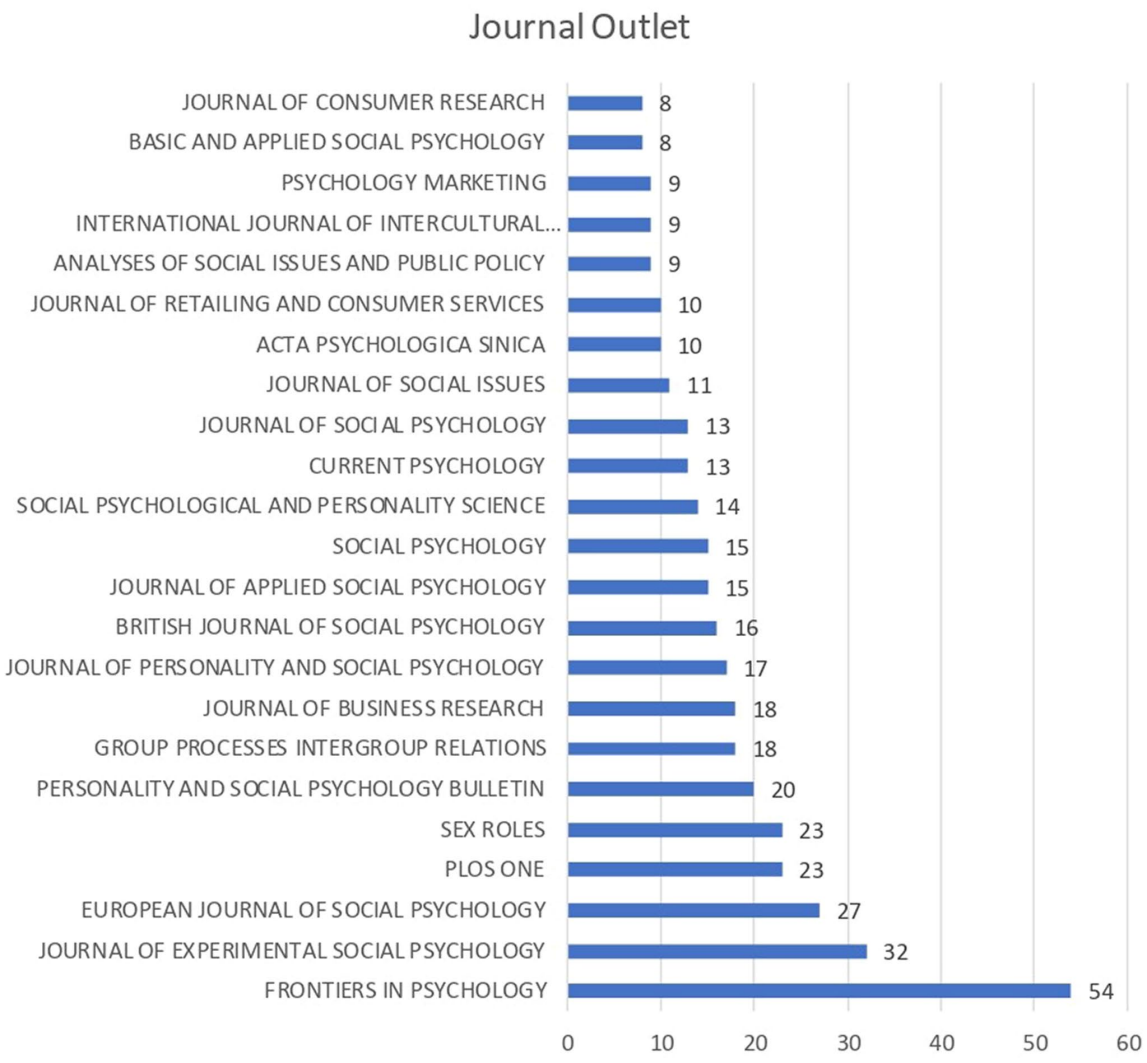
Number of articles published in scientific journals.

The journal that published more articles was Frontiers in Psychology (*N* = 56; 5.27%), followed by the Journal of Experimental Social Psychology (*N* = 32; 3.3%), and European Journal of Social Psychology (*N* = 27; 2.8%). In marketing and consumer behavior, the journals that published more articles were the Journal of Business Research (*N* = 18; 1.88%), Journal of Retailing and Consumer Services (*N* = 10; 1%), Psychology and Marketing (*N* = 9; 0.88%) and Journal of Consumer Research (N = 8; 0.8%) (see Figure 3).

**Figure 4:**
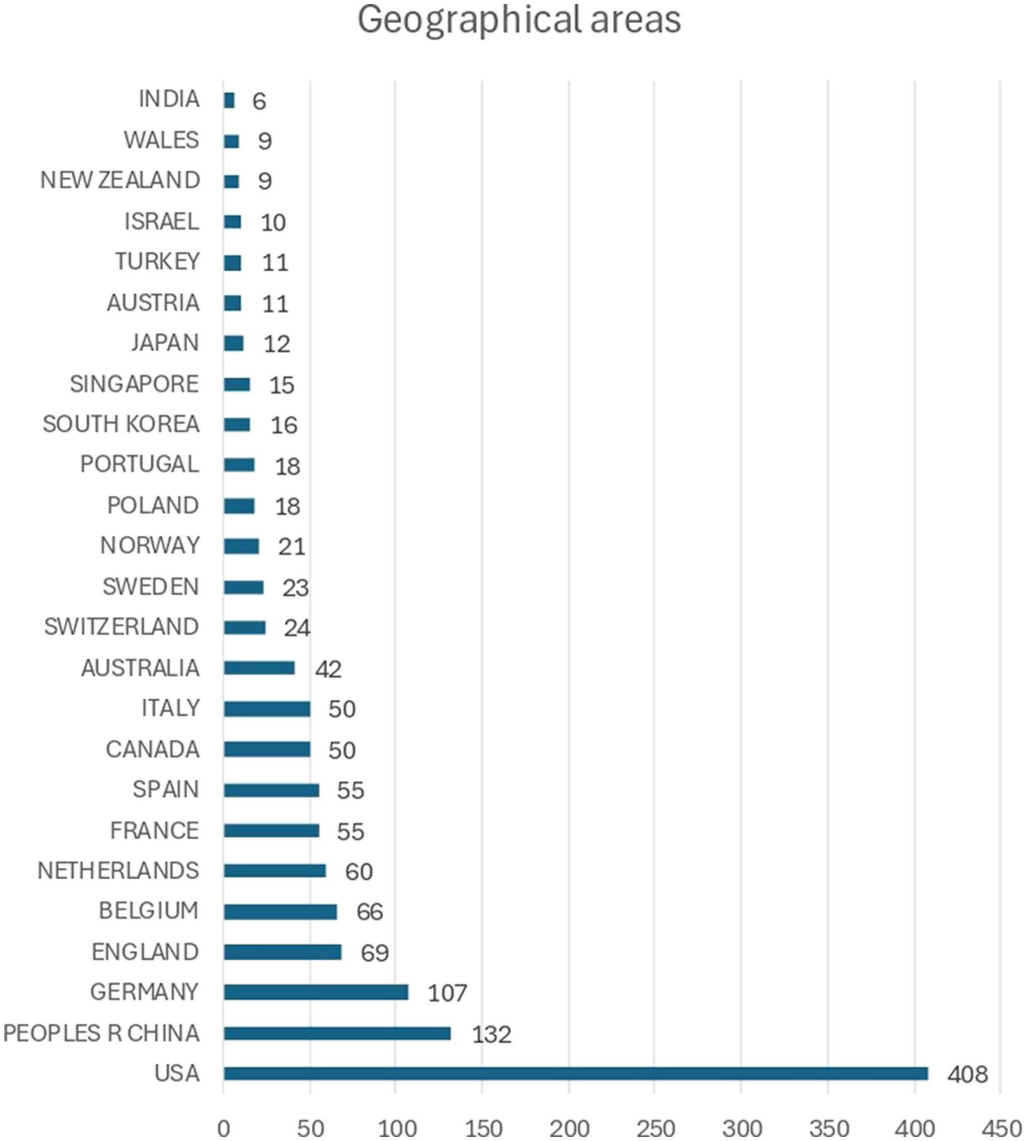
Number of articles per country.

The majority of the studies have been conducted in the United States (*N* = 408; 42.7%), China (*N* = 132; 13.8%), Germany (*N* = 107; 11.2%), England (*N* = 69; 7.2%), Belgium (*N* = 66; 6.9%), Netherlands (*N* = 60; 6.3%), France (*N* = 55; 5.8%), Spain (*N* = 55; 5.8%), Canada (*N* = 50; 5.2%), and Italy (*N* = 50; 5.2%) (see Figure 4). As it can be observed, besides the studies of China, most prior research has been conducted in Western society, namely, North American and European countries.

**Figure 5:**
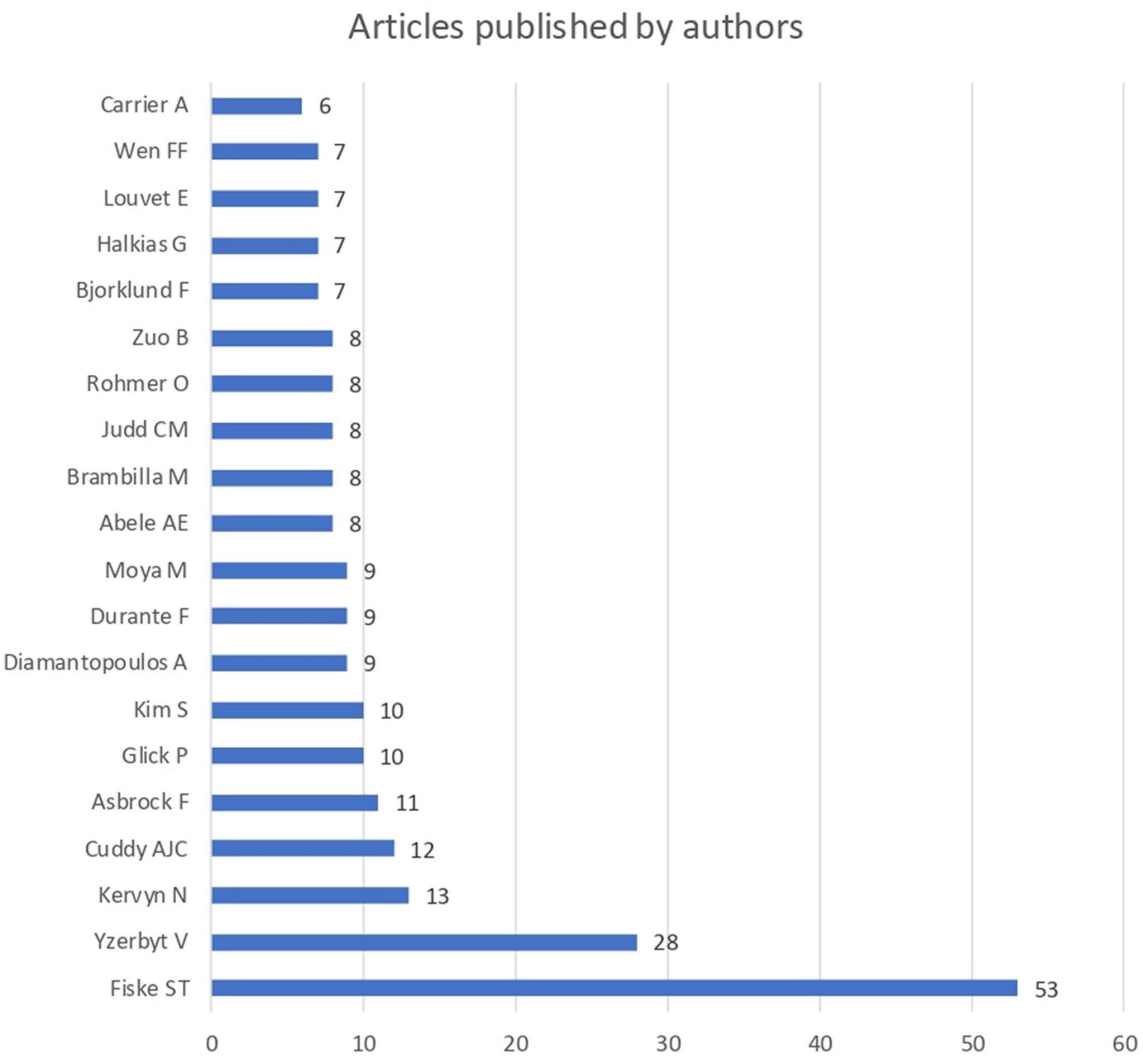
Number of articles per author.

The authors that published more articles were Susan T. Fiske (Princeton University, United States) (N = 53), followed by Vincent Yzerbyt (Université Catholique de Louvain, Belgium) (*N* = 28), Nicolas Kervyn (*N* = 13), Amy Cuddy (Harvard University, United States) (*N* = 12), and Frank Asbrock (Philipps University of Marburg, Germany) (N = 11) (see Figure 5). The studies by Fiske et al. (2002, 2007) were the ones with the higher number of citations (i.e., 4,131, and 2,384 citations respectively), followed by the research by Cuddy et al. (2007, 2008); 1,319 and 1,139 citations respectively), and Judd et al. (2005) (691 citations).

### 3.2 Theories

To identify theories, we examine the abstracts, searching for the keywords “theory” or “model.” In total, 777 articles mentioned a theory or model in the abstract, and 66 theories overall, but some articles used more than one theory. Besides the SCM (*N* = 598), some articles used the agency-beliefs-communion (ABC model; *N* = 34), a model by Koch et al. (2016) that builds on the theoretical foundations of the SCM. This model can be used in research as an alternative to the SCM when examining how individuals mentally organize and categorize different social groups. The authors suggest that individuals spontaneously organize social groups based on their agency/socioeconomic success (A), and on their conservative–progressive beliefs (B). Communion (C) (i.e., caring for others, involving qualities such as benevolence, cooperativeness, and empathy) was not found as a dimension on its own, but rather as an emergent quality in the two-dimensional space of A and B.

**Figure 6:**
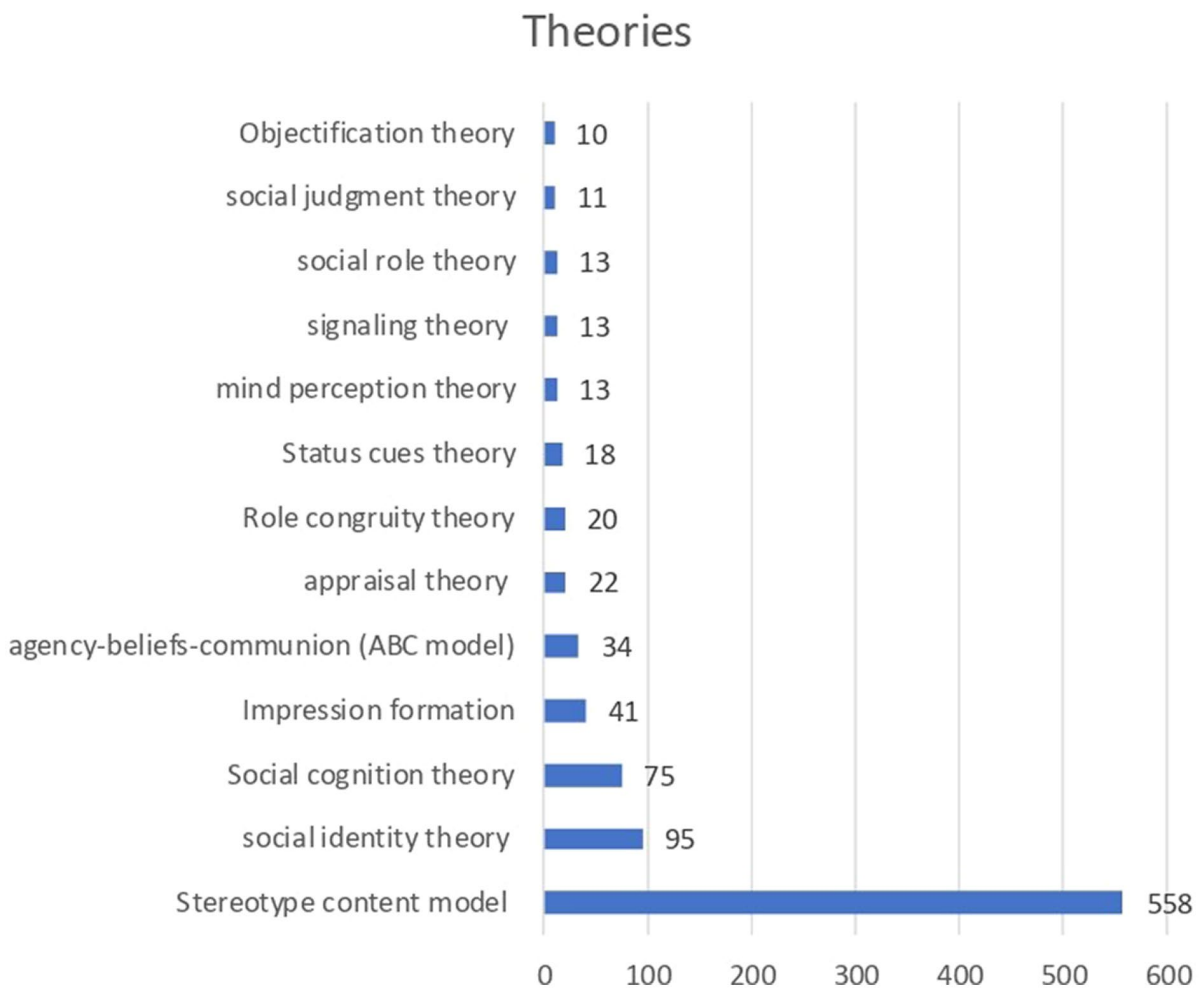
Theories used in published articles focused on the SCM.

Other theories used were the social identity theory (*N* = 95), social cognitive theory (*N* = 75), impression formation theory (*N* = 41), appraisal theory (*N* = 22), and role congruity theory (*N* = 20) (see Figure 6). These theories are highly connected with the SCM, since how people identify themselves with others (identity and congruity), their interaction and impression (cognition and impression formation), as well as the emotions felt from these interactions (appraisal theory), are some important relationships examined in prior research on W/C.

## 4 Themes from bibliographic coupling

We carried out a bibliographic coupling analysis of documents to depict the evolution of scientific knowledge on the SCM over the years. The bibliographic coupling technique was introduced by Kessler (1963), who stated that the articles that refer to common references deal with a similar theme. Bibliographic coupling differs from co-citation. The latter connects articles based on citations received. Thus, to draw knowledge base of a research area, bibliographic coupling can serve better at identifying recent trends (Agostini and Nosella, 2021). In recent years, it has been indicated that this technique is more effective and accurate than co-citation analysis in representing a research front (Mariani and Borghi, 2019; García-Lillo et al., 2020). As mentioned above, most articles on the SCM are recent (i.e., articles that are recent might have received less citations, not necessarily due to the impact, but due to the time of publication). Hence, we opt for the bibliographic coupling technique.

Regarding the software to perform the analysis, we used VOSviewer (Van Eck and Waltman, 2009). This software has been widely used by scholars to carry out systematic reviews (e.g., Lim et al., 2022; Mariani et al., 2022; Basu et al., 2023). The software was developed by van Eck and Waltman (2009) to aid the creation and visualization of bibliometric maps that are easy to interpret. It efficiently collates literature, establishes the similarities among chosen publications within the parameters, and establishes the significant theme among the publications (Tamala et al., 2022). Unlike other computer tools used for bibliometric mapping, VOSviewer places a premium on graphical representation (Van Eck and Waltman, 2009; Oladinrin et al., 2023). The use of VOSviewer as a bibliometric tool to systematically analyze the literature provides several benefits, including a comprehensive literature analysis that allows us to conduct unprecedented scope investigations, a number of tools for extracting reliable data from a series of units of analysis (Cobo et al., 2011), and a transparent set of results offered with a reproducible rigorous process (Oladinrin et al., 2023).

**Figure 7:**
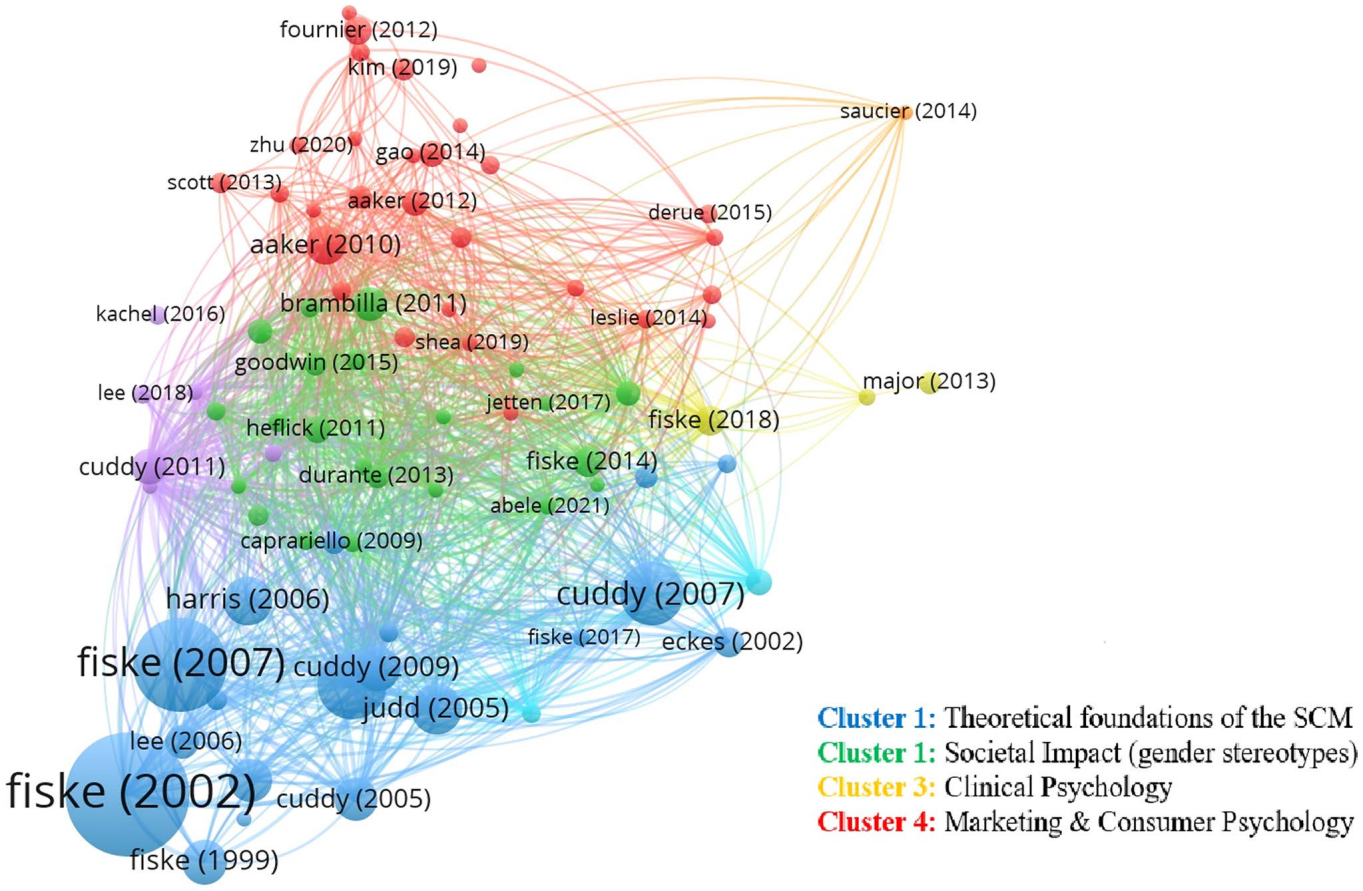
Map from the bibliographic coupling analysis conducted.

Regarding the threshold chosen to perform this analysis, there is some judgment needed in setting the threshold for the minimum number of citations for visualization. A low threshold allows more items to be analyzed and displayed on the map, but also may create overly complex images (Henstock et al., 2022). Following Henstock et al. (2022), whilst VOSviewer applies techniques to optimize the visualization so that labels of nodes do not overlap with each other, for the analysis and interpretation, it was necessary to apply a threshold to limit the number of nodes displayed in the map for clarity. The authors add that applying thresholds is a means to control the VOSviewer’s viewing capabilities, e.g., adjust the number of clusters and nodes displayed to give a clear visualization of the individual nodes and networks. Consequently, the threshold for the minimum number of citations was not determined *a priori*, it was adapted to the number of articles used in connection with good visualization and comprehensive analysis. Using the default option (threshold of 10), the number of authors that appeared was too large to perform a comprehensive analysis and a good visualization. After trying several options, we set a threshold of at least 50 citations per article, leading to a number of authors that allowed us to perform a comprehensive analysis. This produced four clusters. Figure 7 presents the four clusters. The network visualization is color-coded depending on the popularity and similarity of the study. Oppositely, if the color is light, this means that there is a small connection between them.

The analysis shows that the main theoretical foundations by Fiske et al. (1999, 2002, 2007), and Cuddy et al. (2007), provided the essential bases on which all future research built (cluster 1; 23 articles). As observed in Figure 3, this research represents the bottom of a “pyramid,” over which research in social psychology built. This second stream (cluster 2; 12 articles), located over the bottom, examined the societal impact of individuals’ perception of stereotypes of specific groups, with special emphasis on gender stereotypes (e.g., Heflick et al., 2011; Heflick and Goldenberg, 2011). This second stream formed a core of research around which more recent research built. Another research stream identified is associated with clinical psychology and child development (e.g., de Haan et al., 2009; Major et al., 2013; Buist and Vermande, 2014) (cluster 3; 17 articles). Finally, one research stream includes research in the field of marketing (e.g., Aaker et al., 2010, 2012; Zhang et al., 2023) (cluster 4; 22 articles). The next subsections explore each of these streams in more detail.

### 4.1 Cluster 1: the theoretical foundations of the SCM

The most important contribution by Fiske et al. (1999, 2002, 2007), and Cuddy et al. (2007) is that they empirically confirmed the two-dimensional model, forming the basis for a vast amount of future research on stereotypes. Besides providing the main bases, two key assumptions in these studies led to debate and key implications in different fields.

#### 4.1.1 An ambivalent association between the two traits

The authors found that individuals normally characterize other people as warm but incompetent or competent but cold (Fiske et al., 1999, 2002). Judd et al. (2005) challenged this assumption, finding scenarios in which the two dimensions were either positively or negatively associated, while the future work by Fiske et al. (2007) and Cuddy et al. (2009, 2011) supported their earlier propositions. Other research showed that the halo effect and the context can change this direction (Yzerbyt et al., 2005, 2008). This led to research in different contexts, in which conclusions varied (Kervyn et al., 2008, 2015; Sweetman et al., 2013). With all, it has not been fully studied in which scenarios there is an ambivalent or non-ambivalent interaction between the two fundamental traits of social judgment.

#### 4.1.2 A primacy of warmth over competence

Fiske et al. (2002, 2007) argued that individuals try to determine others’ intentions first and, subsequently, their ability to enact those intentions. They added that whether a person is beneficial or harmful (warmth) needs to be inferred first, and then how much benefit/harm the person might cause (competence). Some authors found two key dimensions of this trait, namely, sociability (e.g., friendliness and likeability) and morality (e.g., honesty and trustworthiness) (e.g., Leach et al., 2007). Authors found that morality is more important than sociability (and, as a result, than competence).

### 4.2 Cluster 2: social judgments and prejudices

The SCM has been widely used to examine women’s stereotypes. Using the objectification theory, the work by Heflick and Goldenberg (2011) and Heflick et al. (2011) showed that women (but not men) are perceived as lower in competence and warmth when participants focus on their appearance. This occurs because concentrating on appearance leads to dehumanization (Heflick et al., 2011). Humanization can be associated with honesty, leading to different impressions about others, and different degrees of trust (Brambilla et al., 2012). Another vein studied in social psychology is how competence leads to the perception of higher status, which leads to different types of social representation (Fragale et al., 2011; Dupree and Fiske, 2019), including how warmth can lead to the perception of less successful people (Koch et al., 2016), with lower self-esteem (Wojciszke et al., 2011), but more ethical (Stellar and Willer, 2018).

### 4.3 Cluster 3: application to clinical psychology and child development

Scholars have applied the SCM to mental health, first because stigmatization based on stereotypes can influence serious psychological problems among vulnerable groups (Major et al., 2013). Many of these studies have focused on child development, capturing the perception of siblings (Buist and Vermande, 2014) and parents (de Haan et al., 2009) and how these two traits affect impression and relationship formation, as well as children mental health and behavior (Major et al., 2013).

### 4.4 Cluster 4: findings in marketing

**Figure 8:**
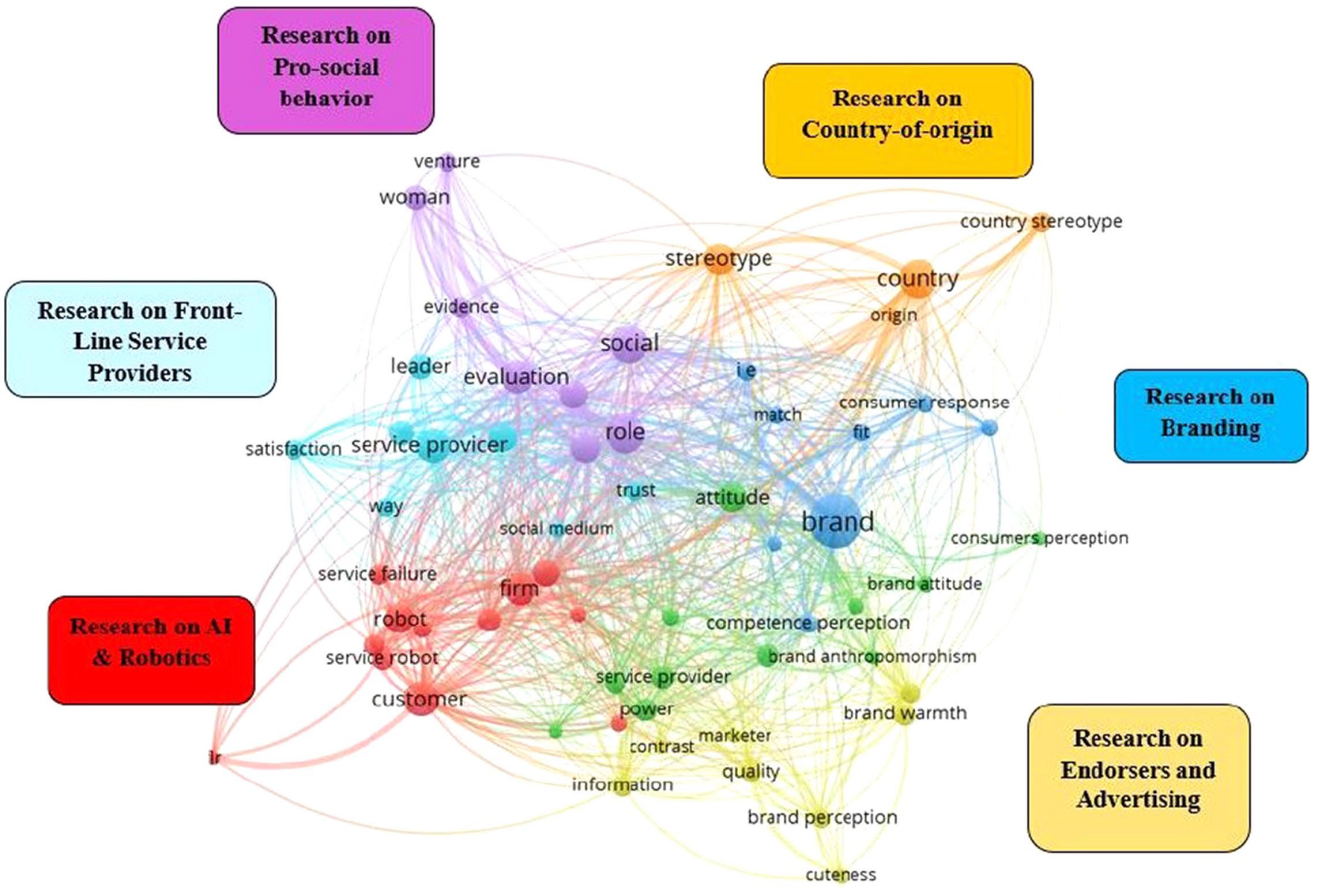
Map from the co-occurrence analysis conducted.

Recent scholars in marketing that have used the SCM have mentioned the need for further research in this field (Leung et al., 2023; Pizzi et al., 2023; Ren et al., 2023). The authors indicated that, while the influence of the two main traits has been more widely studied in psychology, more research is needed to uncover how these traits influence consumers’ decision-making (Kolbl et al., 2019, 2020; Diamantopoulos et al., 2021; Gidaković et al., 2022) and behavior in the market (Halkias and Diamantopoulos, 2020; Pizzi et al., 2023). Following this need for research, in the present study, we first take a closer look at prior findings in marketing and consumer behavior. To conduct this part, we used co-occurrence analysis, only using the studies in the field of marketing. Some authors mentioned that co-occurrence analysis is useful to identify research topics inside a research stream (Mariani et al., 2022). We used VOSviewer. This software places a premium on graphical representation for co-occurrence analysis as well as for bibliometric coupling (Van Eck and Waltman, 2009; Oladinrin et al., 2023), providing several benefits, including a comprehensive identification of topics that allows to synthesize prior findings, and a transparent set of results offered with a reproducible rigorous process (Mariani et al., 2022; Oladinrin et al., 2023). In this case, the predefined number of co-occurrences (i.e., 10) provided a good visualization map, thus, we opt for this criterion to perform the analysis. Through this process, we identified six research streams: (1) branding, (2) country stereotypes (emphasizing the effect of country-of-origin), (3) perception of providers in front-line service, (4) prosocial behavior, (5) perception of endorsers in marketing communications, and, more recently, (6) perception of AI and robots (Figure 8).

#### 4.4.1 Research on branding

One of the most influential studies that applied the SCM to branding was by Aaker et al. (2012). Building on their prior findings (see Aaker et al., 2010), and conflicting with previous results on the primacy of warmth, they observed a primacy of competence in the context of brands and organizations. This is due to consumer admiration of high-competent brands, which in turn leads to higher buying intentions.

These associations might change among different consumers (e.g., based on demographics), and due to the interaction with other variables, such as the use of humor (Brender-Ilan and Reizer, 2021; Howe et al., 2023) or emoticons when communicating the brand (Ivens et al., 2015). Other studies examined the interaction of W/C with the type of narration used to present the brand (i.e., in first vs. third person) (Chang et al., 2019). By examining the interaction of W/C with gender cues, Hess and Melnyk (2016) found that for high-competent brands, feminine cues enhance purchase likelihood, while masculine cues decrease purchase likelihood.

Davvetas and Halkias (2019) provided key practical implications for international marketing. Their study showed that brands associated with localness-induced warmth led to positive consumer responses, whereas brand globalness-induced competence led to some ambivalent reactions. Thus, while the association and implications of brand localness with warmth stereotypes seem clear, there are some contexts in which the relationship between perceived globalness and competence might harm the brand. In this context, Diamantopoulos et al. (2021) found that consumers transfer the country-of-origin stereotypes to the perception of brands. These findings connected research on branding with another of the most relevant topics examined in the field.

#### 4.4.2 Country stereotypes and country-of-origin (COO)

The first theoretical propositions about the influence of W/C and perceptions due to COO on consumer reactions were suggested by Chattalas et al. (2008). Later, Maher and Carter (2011) empirically showed that both, W/C, positively influence consumers’ admiration toward COO brands, and they were negatively related to consumer contempt. However, there might be a primacy of one of the traits due to country stereotypes or the circumstances of the country. During a crisis, warmth is key for consumer forgiveness toward organizations and COO products (Barbarossa and Mandler, 2020). These relationships have been applied to research in tourism, where authors indicate that, when a country is associated with a warmth stereotype, ads with emotional appeals increase visit intention, while ads with rational appeals are more effective when the country is associated with competence (Grigoryev et al., 2019; Feng et al., 2023). These associations influence a favorable attitude and positive intentions toward traveling, but not toward the overall perception of the country (Rojas-Mendez and Davies, 2023).

#### 4.4.3 Perception of front-line service providers

Scott et al. (2013) showed some important findings on the perception of providers based on W/C. The authors indicated that providers’ and sellers’ conspicuous signals decreased warmth and increased competence inferences. Alongside these results, as well as considering prior findings in marketing (i.e., primacy of competence; Aaker et al., 2012), Kirmani et al. (2017) indicated that these associations change among service providers that are positioned as underdogs. This occurs because consumers feel empathy toward this type of provider, thereby attenuating the dominance of competence.

The perception of the provider changes given the perspective of the consumer. For instance, when providers need to enforce rules, if the action is directed at the consumer, they perceive the provider as lower in both traits. If enforced service rules are directed at others, they perceive the provider as higher in both traits, which increases loyalty (Habel et al., 2017). Other authors found that emotional behavior (warmth) reduces the perceived effectiveness of providers handling queries (Singh et al., 2018). Some inferences have been observed based on how much providers/sellers smile (Wang et al., 2017), the physical aspect (Imhoff et al., 2013), such as baby vs. mature face, or how the two traits are attributed to the provider, by effort (higher in warmth) vs. natural talent (higher in competence) (Leung et al., 2020).

Building on conflicting results on the dominance of W/C shown in some of the above-mentioned studies, Güntürkün et al. (2020) provided evidence of an asymmetric dominance, suggesting that warmth is dominant in driving outcomes that capture relational aspects, which is useful for customer retention. On the other hand, competence is dominant in driving outcomes that capture transactional aspects of the customer-service relationship, which is useful for attraction.

#### 4.4.4 Prosocial consumer behavior

Research on this topic has included the effect of perceived W/C on consumers’ intention to engage, accept prosocial requests, and intention to donate. This research stream is more recent, with the first article published in 2018, when Liu and Lin indicated that consumers downplayed their competence, and social warmth (but not their morals), when they wish to sidestep a prosocial request. Zhang et al. (2019) introduced social influence in this context, indicating that, when it comes to willingness to donate to a social cause, high-power individuals accompanied by a romantic (vs. business) partner, respond more positively to messages signaling warmth. Other research examined the effect of attributing to money the ability to sense (warmth) and capacity to do things (competence) to increase charitable giving, finding a primacy of warmth in this context (Zhou et al., 2019). More recently, Chua et al. (2023) indicated that an authentic green brand is instrumental in increasing customer beliefs about both W/C, leading to positive reactions.

#### 4.4.5 Perception of endorsers in marketing communications

Building on the effect of smiles on perceived W/C (Wang et al., 2017), Chen and Wyer (2020) indicated that big smiles male endorsers lead to the perception of higher competence and status. However, this is not the case for female endorsers, who are perceived as higher in warmth and lower in status when they show big smiles. Considering gender as a key variable to operationalize W/C in this context, Bauer et al. (2022) suggested that the key falls into the congruency of the social judgment between the endorser and the product-ad (e.g., brands positioned as high in warmth with an endorse that shows high warmth). Hence, linking celebrity endorser social judgments with the appropriate type of advertising positively influences consumer responses, for both male and female endorsers.

Some factors might provide the desired congruency (Linne et al., 2022). For instance, informal addressing (e.g., *tú* rather than *usted* in Spanish) is preferred by consumers for warmer brands, whereas formal addressing is preferred for competent brands (Leung et al., 2023). Regarding the interaction of these associations with other stimuli, Philipp-Muller et al. (2023) showed that consumers perceive scientific stimuli in marketing communications as competent but cold, which impacts consumers’ reactions to marketers using science to inform about product attributes.

#### 4.4.6 Perception and responses toward AI and robots

Given the increase of robotics and chatbots in front-line services, recent studies applied the SCM to understand consumer reactions and intentions. Research has shown how the two traits affect different dimensions of perceived value and consumer expectations. Initial findings on this topic indicated that perceived robot’s competence influences utilitarian expectations, while perceived warmth influences relational expectations (Belanche et al., 2021). In addition, female gendering (higher warmth) increases acceptance of AI (Borau et al., 2021). Warmth also increases the willingness to use a robot for hedonic services, while competence increases the willingness to use them for utilitarian services.

The presence (vs. absence) of a human provider during the interaction with service robots seems to be key. The presence of humans influences expected service quality, first-visit intention, and willingness to pay (Yoganathan et al., 2021). More recent research indicated that these effects can be moderated by robot anthropomorphism (Choi et al., 2021; Grazzini et al., 2023; Pizzi et al., 2023). Findings suggest that human presence is needed for service recovery when the robot does not look like a human. Thus, only humanoid robots can recover a service by increasing warmth (Choi et al., 2021). Dwivedi et al. (2023) integrated the SCM with the Elaboration Likelihood Model (ELM) and found that cognitive cues and competence influence recommendation intentions among chatbot users. On the other hand, peripheral cues and warmth significantly contribute to positive experiences encountered during the purchase stage. For a clear view of the data, Appendix A provides a complete description of prior works, including authors, year, the research topic, the dimensions of stereotypes examined, dependent variables in the study, and a summary of findings. Supplementary Table A2 is divided by colors, indicating the main research streams identified.

## 5 General discussion

Prior research on the SCM in the fields of psychology and marketing is recent, with the majority of previous research published in the last few years. This shows an increasing trend, showing an increasing interest among scholars, especially in the field of marketing. In addition, most of the prior research has been conducted in Western countries. Thus, there is a need for research of the application of the theory in other regions. There is a lack of findings on the implications of the SCM in regions such as Africa and South America, where studies are practically non-existent. In addition, based on a recognized need for a structured overview of research on the SCM, the present research aimed to identify the main themes among the different research fields that use the SCM framework. This is linked to our first research question. The results of this research show four main themes, namely, (1) the theoretical foundations of the SCM, (2) the effect of SCM on social judgment and prejudices, (3) its application to clinical psychology and child development, and (4) the application to marketing.

**Figure 9:**
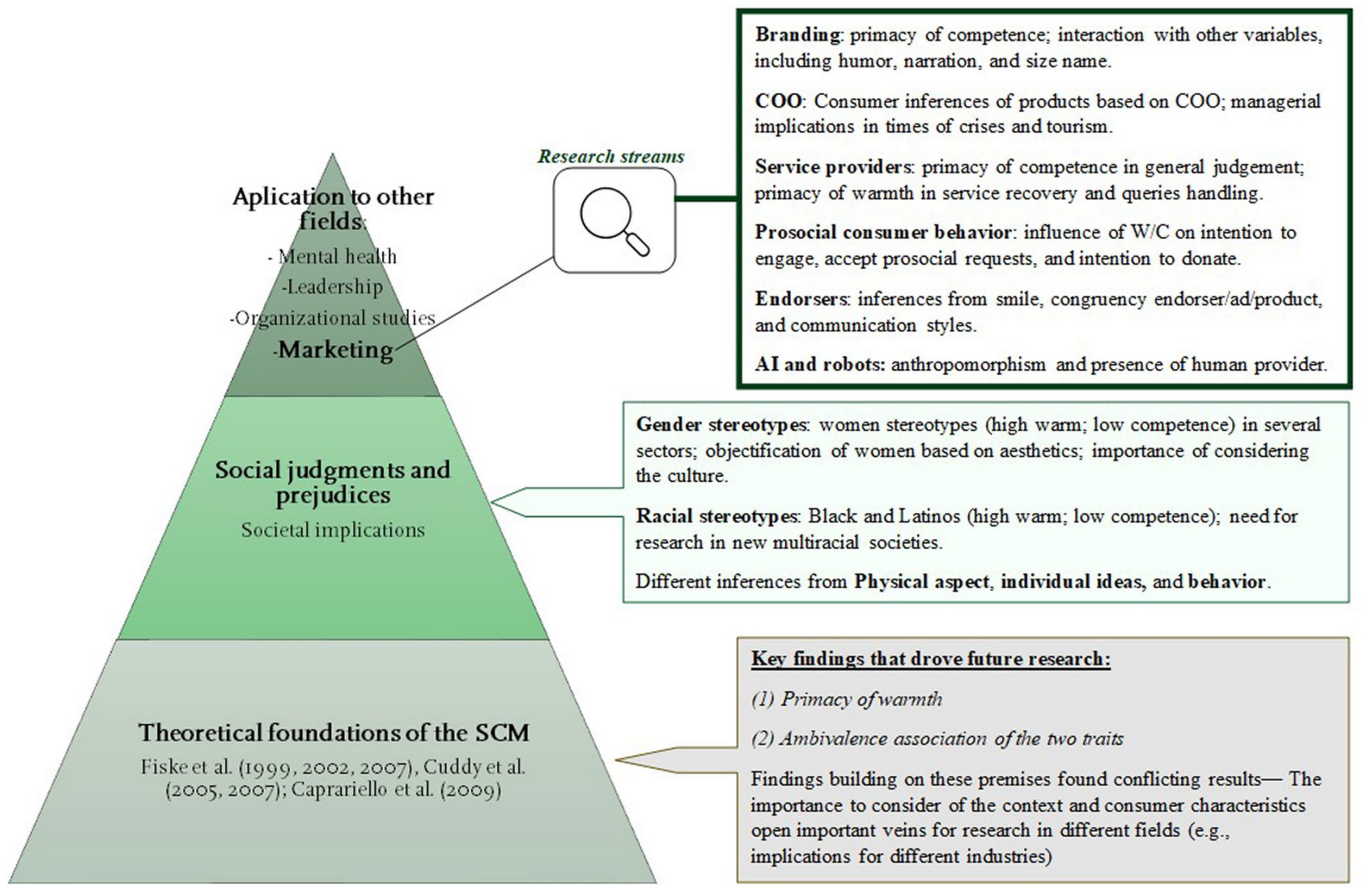
Graphic summary of main topics examined in research that used the SCM.

Finally, in the fourth theme, researchers in marketing explored the effect on brands (e.g., Brender-Ilan and Reizer, 2021), country stereotypes (Grigoryev et al., 2019; Barbarossa and Mandler, 2020; Feng et al., 2023; Rojas-Mendez and Davies, 2023), perception of providers in front-line service (Güntürkün et al., 2020; Leung et al., 2020), the effect of the two traits on consumers’ prosocial behavior (Liu and Lin, 2018; Zhou et al., 2019; Chua et al., 2023), perception of endorsers in marketing communications (Bauer et al., 2022; Linne et al., 2022; Philipp-Muller et al., 2023), and perception of AI (Belanche et al., 2021; Borau et al., 2021; Yoganathan et al., 2021). To summarize the main themes, providing a visual answer to our first research question, Figure 4 provides a graphic summary of the different topics and research streams identified during this review (Figure 9).

### 5.1 Theoretical implications

In this section, we provide the main theoretical implications among the research streams identified. Regarding the theoretical foundations of the SCM (i.e., first theme), research showed that, depending on the context (e.g., sector, industry, etc.), there might be an ambivalent association between the main two traits (Fiske et al., 1999, 2002). However, this assumption was challenged later by other authors (Judd et al., 2005; Richetin et al., 2012), due for example to the influence of the halo effect in some contexts. Similarly, although initially it was assumed the primacy of warmth over competence (Fiske et al., 2002, 2007) other authors inform of the primacy of competence in some contexts, such as the importance of competence when consumers evaluate products and brands (Aaker et al., 2010, 2012; Kolbl et al., 2019, 2020). It can be concluded that the contexts play a key role when examining the direction and primacy of the two main traits that govern social judgments. When examining individuals’ expectations and outcomes in different sectors, research questions should consider the primacy and direction of the two fundamental traits (e.g., ambivalent vs. non-ambivalent associations, inferences from warmth to competence, and *vice-versa*). It is important to provide knowledge regarding which trait individuals consider first in different contexts or for different products, what they expect to find in a particular situation, and which trait influences more positive outcomes during different steps of the consumer experience (e.g., arrival/first contact vs. loyal customers who know the providers well). Due to the context, time, or situation, individuals might infer higher warmth from highly competent professionals, lower competence from highly warm providers, etc. Hence, these two premises need to be considered when examining stereotypes in specific countries (Diamantopoulos et al., 2021; Gidaković et al., 2022), industries, for vulnerable groups, and considering recent changes in current stereotypes (e.g., sexual identities, racial stereotypes, migrant circumstances such as refugees, etc.).

Regarding the second theme, research has shown how individuals evaluate appearance (Heflick et al., 2011) physical aspects, and demographics (Garay et al., 2023) to judge whether a person should—or is more or less likely to—be warmer or more competent. This leads to different reactions of individuals towards certain groups (Adamczyk and Maison, 2023; Castro and Rosa, 2023; Kranz, 2023), which in some cases can be associated with stigmatization (Heflick et al., 2011). Patients who suffer from mental issues can also be stigmatized (Major et al., 2013), which connects with the third theme identified. In the third theme, associated with clinical psychology, research showed how people who face mental health problems use the same judgments as individuals who have not been diagnosed with mental issues (Major et al., 2013). In addition, patients use the same two traits to create impressions about physicians, which can affect their reactions during therapy. This research forms essential basis on how individuals form impressions, which should be used in theoretical frameworks in social sociology, but also in the field of marketing.

Among the four streams identified, recent authors had mentioned the emerging interest among scholars in the SCM and its implications for the fields of marketing (Ren et al., 2023) and consumer research (Leung et al., 2023). Following this need for research we aimed to take a closer look at prior findings in marketing and consumer behavior, which linked to our second research question: Which are the most important topics to explore in future research in marketing? Future research needs in marketing are discussed next.

### 5.2 Future research in marketing

Particularly in the field of marketing, there is a need for research to identify the effect of W/C subdimensions. Besides the distinction between morals and sociability, which has not been explored with as much depth as in social psychology, there are other distinctions that can be important for some industries. For instance, Leung et al. (2020) differentiated between competence due to personal talent and due to effort. Future research should measure the effect of being perceived as hard-working and being perceived as intelligent among service providers, sellers, endorsers, and brands. Findings on the effect of these subdimensions will provide very specific managerial implications. To plan research in this direction, the study by Halkias and Diamantopoulos (2020) provided “unambiguous wording” that can be used to craft new measurements.

Next, we propose a series of topics that need further exploration, due to their importance in the current market and the possibilities of the SCM to provide useful implications (Kolbl et al., 2019, 2020; Diamantopoulos et al., 2021; Gidaković et al., 2022). First, we discuss key veins for future research in connection with the six streams identified (see full review of future research in Supplementary Table A3). As observed in the review of future research among the different streams, the most recent research indicates a need for field studies to validate prior findings in real-life settings. This is an important gap in the literature. As mentioned by Kolbl et al. (2019) cross-cultural studies can add value to the field. Thus, more research in this direction can add value to the field.

Among other possibilities, future research should differentiate between typical (e.g., Mercedes and Germany) vs. atypical (e.g., Red Bull and Austria) COO brand associations; examine how vulnerable consumers respond to ads from competent brands; perform longitudinal studies to observe how, in some sectors, W/C expectations might shift; and examine the antecedents of scarcity (e.g., naturally occurring vs. deliberate on the part of the service provider) to observe W/C inferences.

In the field of robotics and AI, future research should measure different combinations of robots’ W/C, and how the desired features can be achieved while controlling costs, and thus recommend higher efficiency in the market. Future research should also test the effects AI–customer different interaction modalities (e.g., text-only vs. voice-only vs. text and voice), as well as social crowding during the interaction, service encounter duration, control for users’ technological competence, and investigate how social robots can be implemented in situations where there is an externally driven need for a reduction in direct human interaction. All these venues for future research, with the corresponding citation, are described in Supplementary Table A3. Tables can be used by scholars to first identify topics of interest (Supplementary Table A2) and next, to observe which veins for future research authors recommend (Supplementary Table A3).

Finally, we performed a review of all the articles that, although not falling into the six identified streams, provide important bases but have scarcely been explored. To identify these research venues, we carefully read the remaining articles (i.e., those that did not fall into the mentioned six research streams), identified interesting topics and observed that, besides the preliminary findings presented on them, few or no further research have built on them. These topics of research are the following:

#### 5.2.1 Self-perception—influence on motivation and self-efficacy

Research on SCM and self-perception is limited. Initial findings indicated that agency (competence) positively influences self-esteem, while communion (warmth) showed no association with this individual difference (Abele et al., 2016; Abele and Hauke, 2018). More recent findings indicate that warmth is associated with relational self-esteem (Hauke and Abele, 2020). More research is needed in this direction. Future studies should differentiate between morals and sociability, and between talent and effort. Exploring the effects of these subdimensions, from a self-perception point of view, can bring important implications to understand individuals’ motivation, as well as people’s approaches during interactions in different environments (“I am good” vs. “I am nice”). For example, in a buying setting, at work, and during a negotiation. Theoretical models should include self-efficacy as a dependent variable. Relying on self-efficacy theory, we propose including different types of optimism (dispositional vs. unrealistic) as moderators, and self-confidence as a potential mediator, especially among salespeople and consumers, but also in other contexts, such as examining students’ motivation.

#### 5.2.2 Image on social media and the effect on self-perception

One of the most important tools for self-presentation nowadays is social media (Livingston et al., 2020). The way individuals perceive others based on stereotypes influences users’ emotions and behaviors, such as the content consumers generate and the information they share. In turn, this has repercussions for associated brands and products shown during self-presentation (e.g., clothes, tourism destinations in the pictures, etc.). An important factor that has been examined using the SCM is body weight (Adamczyk and Maison, 2023; Castro and Rosa, 2023; Kranz, 2023). The combination of the two topics (i.e., social media and body weight stereotypes) has scarcely been examined in the literature (Livingston et al., 2020). Livingston et al. (2020) found that the idealized woman in social media is perceived as less warm, but equally moral and competent. We encourage research to build on these findings, including other stereotypes besides weight (e.g., racial), and how it affects young individuals’ perception of others and products in the metaverse.

#### 5.2.3 Body size and food stereotypes

When focusing on body size, research should explore cross-cultural variables and societal beliefs (e.g., masculine eating beliefs by men in South America; Luna-Cortes and Aristizabal-Cuellar, 2022), and the effect on eating habits and perception of food. In fact, research on food stereotypes is limited as well, both in clinical psychology and marketing. With all, we encourage scholars to explore how both body weight and food stereotypes (e.g., salad vs. red meat among men vs. women), with implications for consumers’ attitudes toward food and society (e.g., consuming unsustainable products; effects on binge eating, which can lead to obesity).

#### 5.2.4 Research in business-to-business (B2B)

Most research in marketing has focused on consumer outcomes, while fewer studies have examined client organizations (Crisafulli et al., 2020, 2022). Prior research on B2B has found a general primacy of competence. We propose that more research is needed on the perception of the negotiators when it comes to big agreements. Negotiators use perceived tactics differently based on their personality (e.g., negotiators who rate high agreeableness perceive negotiation tactics as unethical) (Skandrani et al., 2021). Future research should examine how different negotiation tactics lead to levels of perceived W/C, the interaction with different personalities, and other individual differences. This will help to further understand organization clients’ perception of B2B brands as well as approaches and outcomes during negotiations.

#### 5.2.5 Innovation and creativity

Only a few studies have examined the effect of W/C on innovation by companies or employees (Kim et al., 2023). Future research should explore co-innovation and value co-creation. Collaboration for innovation has a positive impact on firm growth, and on consumers’ satisfaction, attachment, and loyalty toward the brand. Little is known about how W/C perceptions of the company (or firm representatives, such as managers and endorsers) can affect motivation and the type of ideas consumers provide during co-creation. Future research can also examine individuals’ judgment of employees and other consumers during collaboration for co-innovation.

#### 5.2.6 Stereotypes beyond humans—behaviors toward animals

The studies by Sevillano and Fiske (2016, 2019) showed that W/C judgments predict specific emotions and behavioral tendencies toward animals. No research has built on this venue. Individuals might perceive a pet based on human characteristics (e.g., a member of the family) or an object (e.g., valuing aesthetic characteristics). SCM theory posits that W/C evaluations can lead to humanization or objectification (Heflick et al., 2011). This might have repercussions on the owner-dog relationship, influence educational approaches, and even a serious worldwide problem— relinquishment. However, little is known about how the two traits influence perceived values and pet acquisition. For example, acquiring a dog based on functional values might lead to objectification, while a high emotional value might lead to humanization, influencing W/C perceptions. Questions remain about the empirical evidence of these and similar associations in the context of animals.

#### 5.2.7 Perception of political candidates

Prior research has examined how citizens infer W/C from political candidates, and how this affects image and voting intention. Prior research has focused on appearance and mainly considered the traditional stereotypes based on gender, ethnicity, and age (e.g., Fiske, 2019; Unkelbach et al., 2023). Additionally, prior studies have mainly focused on the US, considering Democratic vs. Republican to measure citizen’s expectations and judgments. Since the perception of liberals, conservatives, and labor parties, among others, might differ among countries, we suggest future research in different regions. Studies should focus on current global problems and the political figure that presents the ideas to approach controversial issues. For instance, in Spain, there are citizens that show different national identities (e.g., in Catalonia, Basque Country). How political figures represent different national identities can affect social judgments toward others inside the same country. For example, based on the language they speak, their behavior, and the products they consume. Other examples can include Arabic political representatives in Western countries (e.g., France) and the citizens’ reaction toward associated ethnic groups in politics.

#### 5.2.8 Research in luxury

Changes in the market have led to a rise in luxury consumption, especially among targets who traditionally did not consume luxury (Luna-Cortés et al., 2022b). A question remains about what consumers expect and value more in terms of W/C for luxury products (see the study by Septianto et al., 2022, in advertising of luxury products). This venue remains practically unexplored. We encourage scholars in tourism and hospitality to build research in this direction, as this is one of the most important luxury industries in terms of market share. For a hotel to be described as luxurious, it needs to offer excellent services by highly qualified providers. Findings in the domain of the SCM show that competence is associated with providers’ capability to provide this kind of service. Research shows that capability concerns reflect uncertainty about the service provider’s ability to accomplish the task (Güntürkün et al., 2020), which is something consumers expect to avoid in a luxury hotel. On the one hand, some research has indicated that being warm may dilute or divert providers’ attention from focusing on properly providing an excellent service (Luna-Cortés et al., 2022b). On the other hand, in the context of non-luxury/affordable hotels, consumers usually show higher concern with price than with the quality of the service. Guests expect service providers at the destination to be helpful and friendly, which can contribute to overcoming skepticism (Luna-Cortés et al., 2022b). These prior associations might help to propose hypotheses on consumers’ expected W/C characteristics in luxury vs. non-luxury experiences.

## 6 Limitations and conclusion

This study presents some limitations. The first limitation is associated with the selection criteria. We included only empirical articles, namely, papers that reported either qualitative or quantitative studies. We presented prior results based on these studies. The use of different methods might lead to different results, and we did not differentiate this when reporting prior findings. For instance, when using some methods (e.g., questions, scales) to measure stereotypes, social desirability bias might play an important role. In addition, there is a key aspect when assessing stereotypes in research, namely, researchers usually need measure stereotype through a third-person technique. These biases should be considered in future research and theoretical frameworks on the SCM. The second limitation is associated with the technique used to perform the analysis. Bibliometric approaches cannot account comprehensively for the complex nature of citing behavior, since it does not capture the rationale behind why authors refer to other works (Agostini and Nosella, 2021). The thrid limitation is associated with the selection of the terms used for the search. Although the selection was based on a thorough conceptual review, the different types of literacy in the literature can lead to debate regarding different keywords used to find all possible constructs. Fourth, the relationships of variables presented in the synthesis of prior findings are based on studies that used different statistical methodologies. It could be argued that differences in methods and analyses could lead to different interpretations of the relationships among the constructs. In addition, we only used articles that were written in English. There might be studies published in marketing in other languages that were not considered in this review. Conference proceedings, reports, and working papers were not considered. Despite these limitations, this research has provided a complete framework integrating together extant research findings. Several research opportunities exist to uncover the particularities of individuals’ behavior in different contexts. Hence, more research is needed to find the direction and interaction of the two traits that form human judgment, as well as the effect of other variables that can have important implications for several industries.

## Data availability statement

The original contributions presented in the study are included in the article/Supplementary material, further inquiries can be directed to the corresponding author.

## Author contributions

GL: Conceptualization, Data curation, Formal analysis, Funding acquisition, Investigation, Methodology, Project administration, Resources, Software, Supervision, Validation, Visualization, Writing – original draft, Writing – review & editing.
