# Supplementary material for: A systematic literature review of the stereotype content model in the fields of psychology and marketing: main themes examined in the literature and an agenda for future research in marketing
Source: Front Psychol. 2024 May 20;15:1392629. doi: 10.3389/fpsyg.2024.1392629 (PMC11144869; doi:10.3389/fpsyg.2024.1392629)
Supplement: Supplementary file 1 [file Presentation_1.zip › Appendix_April 26.docx]

| **Table A1: Set of articles – Bibliographic Coupling** | | | |
| --- | --- | --- | --- |
| ***Cluster 1: The theoretical foundations of the SCM (23)*** | ***Cluster 2: Social judgments and prejudices (12)*** | ***Cluster 3: Application to clinical psychology and Child Development (17)*** | ***Cluster 4: Findings in marketing (22)*** |
| Cameron et al. (2011)  Caprariello et al. (2009)  Cuddy et al. (2005)  Cuddy et al. (2007)  Durante et al. (2013)  Fiske et al. (1999)  Fiske et al. (2002)  Fiske et al. (2007)  Kervyn et al. (2008)  Kervyn et al. (2009)  Kervyn et al. (2010)  Kervyn et al. (2013)  Kervyn (2015)  Leach et al. (2007)  Oldmeadow et al. (2010)  Rohmer et al. (2012)  Rom et al. (2017)  Russell et al. (2008)  Sweetman et al. (2013)  Swencionis et al. (2016)  Vaes et al. (2010)  Yzerbyt et al. (2005)  Yzerbyt et al. (2008) | Carrier et al. (2014)  Dupree et al. (2019)  Fragale et al. (2011)  Goodwin et al. (2014)  Goodwin et al. (2015)  Heflick and Goldenberg (2009) Heflick et al. (2011)  Howe et al. (2019)  Imhoff et al. (2013)  Kervyn et al. (2012)  Koch et al. (2016)  Stellar et al. (2018)  Wojciszke et al. (2011) | Abele et al. (2016)  Altschult et al. (2016)  Buist et al. (2014)  Chen et al. (2014)  De Hann et al. (2009)  Eiden et al. (2016)  Farruggia et al. (2004)  Fiske (2018)  Lansford et al. (2018)  Leung et al. (2010)  Livas-dlott et al. (2010)  Major et al. (2013)  Oldmeadow et al. (2007)  Scaramella et al. (1998)  Spinrad et al. (2007)  Taylor et al. (2010)  Taylor et al. (2015)  Zhou et al. (2002) | Aaker et al. (2010)  Aaker et al. (2012)  Bambrilla et al. (2011)  Barbarossa et al. (2018)  Bennett and Hill (2012)  Borau et al. (2021)  Chang et al. (2019)  Chattalas, et al. (2008)  Davvetas and Halkias (2019)  Diamantopoulos et al. (2021)  Derue et al. (2015)  Fournier et al. (2012)  Gao et al. (2014)  Hess and Melnyk (2016)  Jetten et al. (2017)  Kim et al. (2019)  Kirmani et al. (2017)  Maher and Carter (2011)  Scott et al. (2013)  Shea et al. (2019)  Wang et al. (2017)  Zhu et al. (2020) |

**Table A1. Research in Marketing and Consumer Psychology**

|  | Research in branding (includes perception of organizations) |
| --- | --- |
|  | Country stereotypes (including country-of-origin) |
|  | Perception of providers in front-line service |
|  | Prosocial behavior |
|  | Perception of endorsers in marketing communications |
|  | Perception of artificial intelligence (AI) and robots |

| **Authors** | **Year** | **Topic of research** | **Stereotype dimensions** | **Dependent variables** | **Summary of Findings**  **(*W*/*C:* Warmth; Competence)** |
| --- | --- | --- | --- | --- | --- |
| Chattalas, et al. | 2008 | Country of origin (COO) | Warmth, Competence | Perceived product characteristics (hedonic versus utilitarian); Type of contact service (high‐ vs. low‐contact) | Theoretical propositions (no empirical findings): product’s and consumer’s characteristics determine the effect of W/C on CCO |
| Aaker et al. | 2010 | Non-profit firms | Warmth, Competence | Willingness to buy from non-profit (vs. for-profit). | Non-profit firms are perceived high in W and low in C. Willingness to buy from them (vs. for-profit) is lower. Boosting C in non-profit firms mitigates this effect |
| Maher and Carter | 2011 | Country of origin (COO) | Warmth, Competence | Contempt;  Admiration | W/C are positively related to admiration but negatively related to contempt |
| Aaker et al. | 2012 | Branding | Warmth, Competence | Admiration; purchase intent | The authors support the primacy of competence in the context of organizations and brands. Admiration mediates the relationship between brand competence and purchase intent |
| Bennett and Hill | 2012 | Branding | Warmth, Competence | Purchase likelihood | The authors add that demographic differences impact interactions of consumers with brands |
| Fournier and Alvarez (2011) | 2012 | Branding | Warmth, Competence | N/A | Review: authors propose different veins to advance branding research using the SCM |
| Scott et al. | 2013 | Conspicuous symbolism | Warmth, Competence | Warmth/Competence Inferences;  Attitude; Behavioral intentions toward the provider/seller | Provider’s/Seller’s conspicuous consumption (or wealth signals) decreases warmth  and increases competence inferences |
| Chen et al. | 2014 | Country-related affect (CRA) | Warmth, Competence | CRA | CRA systematically influences product evaluations depending on the valence as well as the warmth or competence associations of CRA |
| Ivens et al. | 2015 | Branding (brand stereotype) | Warmth, Competence | Emotional reactions;  Brand attitudes;  Behavioral intentions | Brand stereotypes mediate the relationships between brand personality and consumers’ brand emotions |
| Dubois et al. | 2016 | Perception of communicators (in low-power vs. high-power states) | Warmth, Competence | Power; Persuasion | High-power communicators generate messages with greater competence information, (which persuades high-power audiences). Low-power communicators generate messages with greater warmth information (persuading more low-power audiences) |
| Hess and Melnyk | 2016 | Branding (gender cues) | Warmth, Competence | Purchase likelihood | For high competence brands, feminine cues enhance purchase likelihood; masculine cues decrease purchase likelihood.  For low competence brands, masculine cues enhance purchase likelihood |
| Kirmani et al. | 2017 | Perception of service providers | Competence  Moral | Consumer preference (i.e., likelihood to choose a service provider) | For service providers, consumers value competence more than morality (underdog positioning moderates this effect) |
| Habel et al. | 2017 | Perception of service providers:  when providers enforce service rules (ESR) | Warmth, Competence | Perceived W/C;  Loyalty | ESR directed at others -perception of high competence and loyalty.  ESR directed to them: low Warmth and low Competence, leading to lower loyalty |
| Lepthien et al. | 2017 | Customer demarketing (dismissing unprofitable customers) | Warmth, Competence | Consumer approval;  Consumer perceived fairness | Consumers perceived fairness if demarketing “is common”, there are financial causes and alternative offers are provided. Consumers find higher/lower fairness dependent on the levels of W/C manipulated |
| Wang et al. | 2017 | Perception of sellers based on smiles | Warmth, Competence | Perception of W/C based on the smile intensity | A marketer displaying a broad smile, compared to a slight smile, is more likely to be perceived by consumers as warmer but less competent. Differences can be observe in different contexts (i.e., high vs. low risk contexts; promotion-focused consumers) |
| Barbarossa et al. | 2018 | Country of origin (COO): during product-harm crisis | Warmth, Competence | Blame attributions; Attitudes toward the company’s products | COO W/C (both) leads to more favorable attitudes. W diminishes blame attributions |
| Liu and Lin | 2018 | Prosocial requests | Two dimensions of warmth: sociability and morality;  Competence | Under-representing W/C to refuse requests | People downplayed competence, and social warmth more than moral warmth, to sidestep a prosocial request |
| Singh et al., | 2018 | Perception of service providers: handling queries | Resolving behavior (competence); Relating (warmth); Emoting behavior (warmth) | Trade-offs between resolving behaviors and warmth (relating and emoting behaviors) | The effectiveness of salesperson’s resolving behavior is reduced by the salesperson’s relating and emoting behaviors |
| Chang et al. | 2019 | Branding (consumer-brand interaction in social media) | Warmth, Competence | Number of “likes” got by first- vs. third-person narratives | First-person narration alongside warm images gained more; Third-person narration accompanied by competent images earns more likes |
| Davvetas and Halkias | 2019 | Branding (Perceived brand globalness) | Intention; Ability; Warmth; Competence | Positive brand affect | Localness-induced warmth has uniformly positive affect, whereas globalness-induced competence can both help and harm the brand |
| Li et al. | 2019 | Providers use of emoticons | Warmth, Competence | Perception of W/C. Satisfaction with the service | Customers perceive service employees who use emoticons as higher in warmth but lower in competence. Results vary among communal- vs. exchange-oriented consumers |
| Zhang et al. | 2019 | Prosocial behavior | Warmth, Competence (signaling in message) | Intention to donate; repurchase intention. | Accompanied by a romantic (vs. business) partner, high-power individuals respond more positively to message signaling warmth (vs. competence). This difference is not observed among low-social-power consumers |
| Zhou et al. | 2019 | Charitable giving (effect of money anthromorphism) | Warmth, Competence | Donation intention | When attributing money the ability to sense (warmth) and capacity to do things (competence), enhancing warmth increases charitable giving |
| Leung et al. | 2020 | Perception of service providers: performance due to dedicated effort vs. natural talent. | Warmth, Competence | Type of relationship expected (communal-oriented, exchange-oriented); perceived W/C; Idea provision behavior; Word-of-mouth | Consumers expect communal-oriented relationship when a provider’s competence is attributed to effort; effort (vs. talent) attribution leads consumers to perceive the provider as warmer |
| Li et al. | 2020 | Perception of tour guides | Warmth, Competence | Perception of tour guide (occupational stigma) | In competence news information, positive valence news information can significantly reduce the public's perception of tour guide occupational stigma |
| Godinho and Garrido | 2020 | Perception of providers and products based on the names (using consonant wanderings) | Warmth, Competence | Consumer preferences and perceived value (hedonic, utilitarian). | For professionals traditionally associated with either a warmth or a competence dimension, inward-wandering usernames systematically presents a competitive advantage |
| Güntürkün et al. | 2020 | Perception of service providers | Warmth, Competence | Consumer attraction; perceived performance; emotional bond; consumer retention | Displaying competence is particular effective in driving customer attraction and current operating performance, whereas displaying warmth is better suited to establish strong emotional bonds and drive customer retention. |
| Chen and Wyer | 2020 | Perception of endorsers | Warmth and competence operationalized by gender | Status perception and purchase intention | Smiling males are perceived as higher in status (opposite for females) |
| Belanche et al. | 2021 | Robots in frontline services | Warmth, Competence | Value expectations (utilitarian, relational) | Perceived competence of the robot influences utilitarian expectations; perceived warmth influences relational expectations |
| Borau et al. | 2021 | Robots and AI in frontline services | Warmth and competence operationalized by gender | Acceptance | Female gendering increases acceptance of AI, as well as humanness perceptions in this context |
| Diamantopoulos et al. | 2021 | Branding (COO brands) | Warmth, Competence | Transfer of country’s W/C to brand’s W/C | Country warmth and competence, respectively, impact brand warmth and competence, thus confirming the hypothesized stereotype content transfer |
| Barbarossa and Mandler | 2021 | COO: Corporate crisis | Warmth, Competence | Emotions and intentions toward the brand | Country stereotypes of warmth—not competence—can buffer negative emotions and retaliatory intent toward an offending company |
| Choi et al. | 2021 | Robots and AI in frontline services | Warmth, Competence | Perceived service recovery; Satisfaction. | Consumers are more dissatisfied due to lack of warmth following a process failure caused by a humanoid (vs. nonhumanoid). However, humanoids (but not nonhumanoids) can recover a service failure by themselves via sincere apology, restoring perceptions of warmth. |
| Yoganathan et al. | 2021 | Robots and AI in frontline services | Warmth, Competence | W/C inferences;  Expected service quality;  First-visit intention;  Willingness to pay | In the absence of a human, anthropomorphizing service robots increases W/C inferences, expected service quality, first-visit intention, willingness to pay |
| Aagerup et al. | 2022 | Branding (B2B) | Warmth, Competence | N/A | By focusing on brand competence in the realm of the actual product and brand warmth in the realm of the augmented product, the companies manage to create a complete and consistent brand personality |
| Bauer et al. | 2022 | Perception of endorsers | Warmth, competence, and genders | Consumer responses: choice, ratings and willingness to pay. | Matching celebrity endorser social judgments with the appropriate type of advertising positively influences consumer responses, for both male and female endorsers |
| Liu (B.Q.) and Li | 2022 | Service recovery | Warmth, Competence (use as mediators) in a study using baby (vs. mature) faced providers | Consumer forgiveness; Recovery satisfaction | Baby-faced service provider has a more positive effect on consumer forgiveness and recovery satisfaction. W/C mediate the relationships |
| Liu (X.) et al. | 2022 | Robots and AI in frontline services | Warmth, Competence | Willingness to use robots | Consumers are more willing to use a service robot perceived as warm in hedonic service contexts; they are more willing to use a service robot perceived as competent in utilitarian service contexts |
| Xu and Liu (J.) | 2022 | Robots and AI in service recovery (use of humor) | Warmth, Competence | Consumer response | There is a positive impact of humorous response on customer response. Perceived W/C—along with boundaries (customer inoculation and time pressure)—mediate the effect of humor |
| Zhang et al. | 2022 | Branding (brand names) | Warmth, competence, and genders | Perceived W/C based on name size | Brands with a size cue of smallness in the name are perceived to be warmer but less competent, while those with a size cue of bigness are perceived to be less warm but more competent |
| Chua et al. | 2023 | Sustainable brands | Warmth, Competence | Perceived W/C; Trust; Authenticity; Consumer choice | The study supported that an authentic green brand is instrumental in increasing customer beliefs about its warmth and competence, leading to positive reactions |
| Dwivedi et al. | 2023 | Chatbots in frontline services: combination of Elaboration Likelihood Model (ELM) cues with SCM (W/C). | Warmth, Competence | Intention to recommend; Experience value | Cognitive cues and C influence recommendation intentions among chatbot users. Peripheral cues and Warmth significantly contribute to positive experiences encountered during the purchase stage |
| Feng et al. | 2023 | Country stereotypes | Warmth, Competence | Perceived risk; Travel intention | When a country is associated with a warmth stereotype, ads with emotional appeals increase visit intention. Ads with rational appeals are more effective when the country is associated with a competence stereotype |
| Grazzini et al. | 2023 | Chatbots in frontline services | Warmth, Competence (used as moderators) | Consumer responses; Disconfirmed expectations; Perceived eeriness | Bots’ human-likeness leads to higher negative customers’ responses (mediated by disconfirmed expectations rather than perceived eeriness). However, when customers interact with a warm robot high in human-likeness, this negative effect vanishes |
| Howe et al. | 2023 | Branding (use of humor) | Warmth, Competence (mediators in the model) | Brand attitude; brand engagement | As the cleverness in humor increases, consumers brand attitudes and engagement increases. W/C mediates this reaction |
| Kim et al. | 2023 | Branding (relationship partners) | Warmth, Competence | Brand preferences | Consumers perceive warm (vs. less warm) brands as better relationship partners |
| Kim et al. | 2023 | Consumer interaction with Robots and AI (vs. human) | Warmth, Competence (not part of the main study, but used as control) | Consumer’s unethical behavior | Interacting with non-human (vs. human) agents, such as AI and robots, increases the tendency to engage in unethical consumer behaviors due to reduced anticipatory feelings of guilt |
| Leung et al. | 2023 | Formal vs. Informal addressing by advertisers and service providers | Warmth, Competence | Consumer preferences and responses toward the brand | Informal address preferred when used by warmer brands; whereas formal address is preferred when used by more competent brands |
| Li and Ma | 2023 | Branding (destination logo) | Warmth, Competence | Attitude; Travel intention | Linking logos with handwritten typefaces with warm destinations, and machine-written typefaces with competent destinations can elicit more favorable attitudes and stronger travel intentions |
| Malika and Maheswaran | 2023 | Perception of service firms (brand image) | Warmth, Competence | Perception of W/C; Purchase intentions | Busy service firms are perceived higher on competence and poor service firms are perceived higher on warmth |
| Philipp-Muller et al. | 2023 | Use of science in marketing communications and branding | Warmth, Competence | Perception of W/C; functional and hedonic value; product valuation | Consumers view the scientific process as competent but cold, which impacts consumers’ reactions to marketers using science to inform about their brands/products |
| Pizzi et al. | 2023 | Robots and AI in frontline services | Warmth, Competence | Trust; willingness to disclose personal information; Willingness to re-patronize the e-tailer | W perceptions are affected by gaze direction, whereas C perceptions are affected by anthropomorphism. W/C affects consumers’ trust and behavioral intentions |
| Rojas-Mendez and Davies | 2023 | Country image (in new underdeveloped markets) | Warmth, Competence, Status (independent from competence) | Tourism attitude; country attitude. | Status was not mediated by competence in this context (as prior studies suggested). Warmth evaluations predict tourism attitude, but no country attitude |
| Scott et al. | 2023 | Racial bias in financial services | Warmth, Competence | Service outcomes (products offered); Perceived W/C during the service process; Loyalty intentions | The authors discuss several discriminating situations of Whites toward Blacks in service providers. They indicate in which circumstances such discrimination is mitigated (e.g., Black customer signaling high status) |

***Source: own elaboration***

**Table A2. Future research directions in each research stream**

| **Research stream** | **Study** | **Future directions** |
| --- | --- | --- |
| **Branding** | Diamantopoulos et al. (2021) | - Disentangle the role of typicality in conjunction. This is, including brands rather typical of their COO (e.g. Mercedes and Germany), vs. atypical brands (e.g. Red Bull and Austria). - Study stereotypes of well-known in comparison with COO products from less known countries (i.e., no clear stereotypes). - Research needed in less developed countries. Consumers from less developed countries tend to rely even more on country stereotypes. Results with these samples are limited. - Include the stereotype of the typical brand user and observe interaction with COO. |
|  | Aagerup et al. (2022) | Investigations of how customers evaluate B2B brands, as well as research on B2B brands in other industries.   - Replication studies with different products and industries.   Study how brand personality affects B2B relationships.   - Examine self-image congruity for B2B brands. |
|  | Zhang et al. (2022) | - Examine the extent to which perceived warmth/competence might influence the perceived gender of the brand. - Investigate how the shift in gender roles in some societies interact with gender/size associations. - Examine how, for some products, reverse relationships (e.g., computer chips: smallness might lead to competence). - Explore the effect of size of brand nicknames. |
|  | Howe et al. (2023) | - Examine which types of brands benefit more from clever humor. - Examine contexts in which humor might not be accepted by consumers. |
|  | Kim et al. (2023) | - Test how consumer–brand relationships created via social exclusion predict consumer behavior in different contexts. - Explore how excluded consumers respond to transgressions by warm brands (e.g., service failures), and whether or not these relationships would survive (leading to forgiveness) or be undermined by a transgression. - Examine how excluded consumers respond to promotions from competing brands. - Investigate the importance of warmth among specific targets, such as older consumers or vulnerable population. - Examine other socially adverse contexts such as bullying or explicit rejection. |
|  | Leung et al. (2023) | The authors suggest including several potential effects in future experiments:   - Possible moderators: - Consumer expectations. - Individual differences - Power vs. solidarity. - Communication context. - Brand loyalty. - Additional factors: - The role of cultural values in preference for pronominal address. - Cross-linguistic differences in pronominal address. - Include gender-neutral pronouns. - Other dimensions of personality. - Interaction between warmth and competence. - Include expectation and disconfirmation as a direct effect. - How pronominal address shapes brand personality |
|  | Malika and Maheswaran (2023) | - Examine the effect of scarcity of both money and time resources (such as limited space or service staff). - Examine the hypotheses in different cultures. - Interactions of warmth and competence (e.g., explore whether the perceptions of low competence could have negative spillover effects on warmth perceptions). - Include some antecedents of scarcity (e.g., naturally occurring vs. deliberate on the part of the service provider). - Examine other contexts (e.g., salesforce management, couponing, and online contexts). - Examine how scarcity affect differently consumer-brand relationship and consumer-service firm relationship. |
|  | | |
| **Country of Origin**  **(Country stereotypes)** | Barbarossa and Mandler (2021) | - Include the effect of brand familiarity (using real brands, rather than fictional). - In connection with the above, examine aspects related to brand positioning. - Examine the role of prominent consumer dispositions toward foreign countries (e.g., consumer ethnocentrism, cosmopolitanism, and consumer affinity/animosity). - In connection with the latter, include perceived severity and intentionality as statistical controls and/or moderating variables. - Use of different scales in future replication studies. - Examine the relationships in the context of corporate crises (i.e., include emotional and cognitive responses in this context). |
|  | Feng et al. (2022) | - Replicate the study in Western countries. - Include interacting variables, specifically, brand personality and brand identification. - Observe (potential) discrepancies between local tourism and international tourism. - Investigate the potential impacts of perceptual and linguistic fluency when processing tourism advertising on travel decisions. - Examine benefits of disfluent metacognitive experiences, such as stimulation of curiosity, and examine when fluency can enhance versus hinder the effectiveness of tourism advertising. |
|  | Rojas-Méndez and Davies (2023) | - Explore the relationships in other contexts/industries. - Use new targets and control for similar sample sizes. - Examine deeply effect of interacting variables based on the communication (what is said about the country) and tangible aspects of the country. |
|  | | |
| **Service providers** | Güntürkün et al. (2020) | - Longitudinal studies: how the perception of warmth and competence develop over time. - Use objective measures (rather than self-report) to capture transactional outcomes (e.g., objective sales data). - Investigate the relationships using field experiments. - Test multi-level design to explore differences among different service provider size or marketing strategies. - Examine how the two dimensions (i.e., W/C) conceptually and empirically relate to other evaluative frameworks (e.g., different Service Quality dimensions). |
|  | Leung et al. (2020) | - Examine other contexts (e.g., for different products, firms and brands). - Explore the relationships for service failure or recovery. - Explore how consumers interpret information of firms whose performance is uncertain (e.g., startups). - Include other consumer outcomes in future theoretical models (e.g., preferences, loyalty). - Include the effect (e.g., moderating) of relationship expectations. - Explore consumer heterogeneity in terms of attributions of service employee performance. |
|  | Liu and Li (2022) | - Conduct field experiments. - Cross-cultural studies. - Include moderating variables: work-related variables (i.e., service expertise) or employee-related variables (i.e., gender). |
|  | | |
| **Pro-social behavior** | Zhang et al. (2019) | - Besides cause-related marketing, other forms of corporate social responsibility initiatives should be explored. - Explore different social contexts. - Test the effect on other outcomes, besides donation. - Use field experiments. - Include cross-cultural variables/samples in future analyses. |
|  | Zhuo et al. (2019) | - Examine the link between money warmth and charitable giving. - Explore other outcomes: products that are associated with warmth (e.g., children clothes). - Test the effect of money anthropomorphism on changing behavior. - Explore the antecedents of money anthropomorphism (e.g., uncertainty in behavior, which is link to human characteristics). |
|  | Chua et al. (2023) | - Test the relationships with other products. - Test the effect on brand trust. - Investigate information processing in this context takes place. - Test the effect (e.g., moderating) of consumer skepticism. |
|  | | |
| **Endorser stereotypes** | Chen and Wyer (2020) | - Perform a more systematic analysis of specific product features. - Use real life advertising examples. - In connection with the latter, consider the positioning of the brand. - Include the role of endorsers’ autonomy. |
|  | Bauer et al. (2022) | - Include other types of endorsers (besides actors; e.g., musicians, athletes, etc.). - Test the effect of brand familiarity. - Differentiate the messaging (symbolic vs utilitarian). - Differentiate between the type of endorsement (implicit vs explicit). - Test the effect of consumer involvement. - Test the effect of trust. - Explore the relationships on the Internet (i.e., different online environments). |
|  | Philipp-Muller et al. (2023) | - Explore the source of a lay belief: examine socio-cultural and personal variables. - Use larger samples (this is related to a limitation found in some specific studies). - Retest the effect with non-explicit reference to science (e.g., visual stimuli: a laboratory setting). |
|  | | |
| **AI and robotics** | Liu et al. (2022) | - Perform field experiments, including behavioral variables. - Replicate the findings for embodied service robots. - Include different combinations of robots’ warmth and competence while controlling the costs of achieving effective features (i.e., identify most efficient combinations). - Identify factors that moderate the congruity effect, for instance, travel type (e.g., leisure travel vs. business travel). - Examine the effect of distinctive service types in the same service context (e.g., coffee bar: hedonic-dominant vs. hotel room context: utilitarian-dominant). - In connection with the above, include the moderating role of sense of power. - Explore how trust-related variables influence the effect of congruity on tourists’ behaviors. |
|  | Xu et al. (2022) | - Consider different types of humor to verify whether such humor types influence the recovery effect of humor. - Replicate the findings in other contexts. - Use different samples (from other countries). - Examine the effect of different emotional valence (positive vs. negative) on humorous responses. - The AI–customer interaction modality (text-only vs. voice-only vs. text and voice) can be explored as a moderator. - Other moderators on humorous responses: social crowding; service encounter duration. - Examine service providers’ humorous responses to customers' negative reviews. |
|  | Dawetini et al. (2023) | - Perform longitudinal studies to evaluate and compare between purchase and post-purchase stages. - Deploy a more detailed and robust framework of chatbots to test the cognitive and peripheral routes. - Examine the moderating role of users’ technology competence. - Consider the different aspects of experience, such as cognitive, affective, and conative experience. - Consider service quality dimensions. |
|  | Grazzini et al. (2023) | - Conduct field experiments. - Test the results in other settings, e.g., hotels. - Investigate specific expectations’ dimensions with reference to warmth and competence (e.g., honesty, kindness, trustworthiness efficiency, reliability, responsiveness, accuracy). - Include the moderating effect of the nature of the tasks that the service robot is required to complete. - Investigate how social robots can be implemented in situations where there is an externally driven need for a reduction in direct human interaction. - Perform a deeper investigation of customers’ emotional feelings (e.g., empathy). |
|  | Kim et al. (2023) | - Future research can examine if other individual differences (e.g., attitude toward technology, religious worldview, experience, and familiarity with technologies) can moderate the effects documented. - Identify certain AI design features or decision contexts that can influence the perception of AI’s capabilities to detect fraud. - Examine whether individuals perceive less harm or damage to the firm when the identical unethical behavior was committed to an AI (vs. a human) agent. |
|  | Pizzi et al. (2023) | - Extend our findings by incorporating more aspects related to chatbots’ anthropomorphism. - Replicate the findings by enlarging the chatbot’s set of verbal and nonverbal communication cues (effect on perceived anthropomorphism). - Explore whether different chatbot characteristics (e.g., gaze direction and/or anthropomorphism) alter consumers’ likelihood of starting or leaving a conversation with a chatbot. - Analyze consumers’ actual visual search behavior and emotional reactions to chatbots. |

**References Table A1 and Table A2**

Aagerup, U., Andersson, S., & Awuah, G.B. (2022). Building a warm and competent B2B brand personality. *European Journal of Marketing, 56*(13), 167-193.

Aaker, J., Vohs, K. D., & Mogilner, C. (2010). Nonprofits are seen as warm and for-profits as competent: Firm stereotypes matter. *Journal of Consumer Research, 37*(2), 224–237.

Aaker, J. L., Garbinsky, E. N., & Vohs, K. D. (2012). Cultivating admiration in brands: Warmth, competence, and landing in the “golden quadrant”. *Journal of Consumer Psychology, 22*(2), 191–194.

Brambilla, M., Sacchi, S., Rusconi, P., & Goodwin, G.P. (2021). The primacy of morality in impression development: Theory, research, and future directions. *Advances in Experimental Social Psychology*, *64*, 187-262.

Barbarossa, C., Pelsmacker, P.D., & Moons, I. (2018). Effects of country-of-origin stereotypes on consumer responses to product-harm crises. *International Marketing Review, 35*, 362-389.

Barbarossa, C., & Mandler, T. (2020). Not all wrongdoers are equal in the public eye: a moderated mediation model of country stereotypes, condemning emotions, and retaliatory intent in corporate crises. *Journal of International Marketing, 29*, 26-44.

Bauer, B.C., Carlson, B.D., & Johnson, C.D. (2022). The match-up hypotheses revisited: matching social judgments and advertising messaging in celebrity endorsements. *European Journal of Marketing*, *56*(3), 869-898.

Belanche, D., Casaló, L.V., Schepers, J.J., & Flavián, C. (2021). Examining the effects of robots' physical appearance, warmth, and competence in frontline services: The Humanness‐Value‐Loyalty model. *Psychology & Marketing*, *38*(12), 2357-2376.

Bennett, A. M., & Hill, R. P. (2012). The universality of warmth and competence: A response to brands as intentional agents. *Journal of Consumer Psychology, 22*(2), 199–204

Bernritter, S. F., Verlegh, P. W. J., & Smit, E. G. (2016). Why nonprofits are easier to endorse on social media: the roles of warmth and brand symbolism. *Journal of Interactive Marketing*, *33*(1), 27-42.

Borau, S., Otterbring, T., Laporte, S., & Fosso Wamba, S. (2021). The most human bot: Female gendering increases humanness perceptions of bots and acceptance of AI. *Psychology & Marketing, 38*(7), 1052-1068.

Chang, Y.P., Li, Y., Yan, J., & Kumar, V. (2019). Getting more likes: the impact of narrative person and brand image on customer–brand interactions. *Journal of the Academy of Marketing Science, 47,* 1027–1045.

Chattalas, M., Kramer, T., & Takada, H. (2008). The impact of national stereotypes on the country of origin effect: *A conceptual framework. International Marketing Review, 25,* 54-74.

Chen, C. Y., Mathur, P., & Maheswaran, D. (2014). The effects of country-related affect on product evaluations. *Journal of Consumer Research, 41*(4), 1033–1046.

Chen, Y., & Wyer, R.S. (2020). The effects of endorsers' facial expressions on status perceptions and purchase intentions.*International Journal of Research in Marketing, 37,* 371-385.

Chua, B., Kim, S., Baah, N.G., Moon, H., Yu, J., & Han, H. (2023). When hospitality brands go green: the role of authenticity and stereotypes in building customer-green brand relationships. *Journal of Sustainable Tourism*, Ahead-of-print: https://doi.org/10.1080/09669582.2023.2203406

Davvetas, V., & Halkias, G. (2019). Global and local brand stereotypes: formation, content transfer, and impact*. International Marketing Review, 36*(5), 675-701.

Diamantopoulos, A., Szőcs, I., Florack, A., Kolbl, Ž., & Egger, M. (2021). The bond between country and brand stereotypes: insights on the role of brand typicality and utilitarian/hedonic nature in enhancing stereotype content transfer.*International Marketing Review, 38*(6), 1143-1165.

Dubois, D., Rucker, D. D., & Galinsky, A. D. (2016). Dynamics of communicator and audience power: The persuasiveness of competence versus warmth. *Journal of Consumer Research, 43*(1), 68–85.

Dwivedi, Y.K., Balakrishnan, J., Baabdullah, A.M., & Das, R. (2023). Do chatbots establish “humanness” in the customer purchase journey? An investigation through explanatory sequential design.*Psychology & Marketing, 40*(11), 2244-2271.

Feng, W., Liu, Y., & Li, D. (2022). Emotional or rational? The congruence effect of message appeals and country stereotype on tourists' international travel intentions. *Annals of Tourism Research*, *95*, 103423.

Fournier, S., & Alvarez, C. (2012). Brands as relationship partners: Warmth, competence, and in-between. *Journal of Consumer Psychology, 22*(2), 177–185.

Godinho, S., & Garrido, M.V. (2020). The “ins” and “outs” of product and services marketing: The influence of consonant wanderings in consumer decision‐making. *Psychology & Marketing, 37*, 1352-1361.

Grazzini, L., Viglia, G., & Nunan, D. (2023). Dashed expectations in service experiences. Effects of robots human-likeness on customers’ responses. *European Journal of Marketing*, *57*(4), 957-986.

Güntürkün, P., Haumann, T., & Mikolon, S. (2020). Disentangling the differential roles of W/C judgments in customer-service provider relationships. *Journal of Service Research*, *23*(4), 476-503.

Habel, J., Alavi, S., & Pick, D. (2017). When serving customers includes correcting them: Understanding the ambivalent effects of enforcing service rules. *International Journal of Research in Marketing, 34*, 919-941.

Halkias, G., & Diamantopoulos, A. (2020). Universal dimensions of individuals' perception: Revisiting the operationalization of warmth and competence with a mixed-method approach. *International Journal of Research in Marketing, 37*, 714-736.

Hess, A.C., & Melnyk, V. (2016). Pink or blue? The impact of gender cues on brand perceptions. *European Journal of Marketing, 50*, 1550-1574.

Howe, H., Zhou, L., Dias, R.S., & Fitzsimons, G.J. (2023). Aha over Haha: brands benefit more from being clever than from being funny. *Journal of Consumer Psychology*, *33*(1), 107-114.

Ivens, B.S., Leischnig, A., Muller, B., & Valta, K.S. (2015). On the role of brand stereotypes in shaping consumer response toward brands: an empirical examination of direct and mediating effects of warmth and competence. *Psychology & Marketing, 32*, 808-820.

Kim, S., Murray, K.B., & Moore, S.G. (2023). Some like it warm: How warm brands mitigate the negative effects of social exclusion. *Psychology & Marketing*, *40*(4), 777-790.

Kim, T., Lee, H., Kim, M.Y., Kim, S., & Duhachek, A. (2022). AI increases unethical consumer behavior due to reduced anticipatory guilt. *Journal of the Academy of Marketing Science, 51*, 785 - 801.

Kirmani, A., Hamilton, R.W., Thompson, D.V., & Lantzy, S. (2017). Doing well versus doing good: the differential effect of underdog positioning on moral and competent service providers. *Journal of Marketing, 81*, 103-117.

Li, F., & Ma, J. (2023). The effectiveness of the destination logo: Congruity effect between logo typeface and destination stereotypes. *Tourism Management, 98*, 104772.

Li, X. (S.), Chan, K. W., & Kim, S. (2019). Service with emoticons: How customers interpret employee use of emoticons in online service encounters. *Journal of Consumer Research, 45*(5), 973–987.

Liu, B., & Li, Y. (2022). Teddy-bear effect in service recovery. *Annals of Tourism Research*, *94*, 103400.

Liu, P. J., & Lin, S. C. (2018). Projecting lower competence to maintain moral warmth in the avoidance of prosocial requests. *Journal of Consumer Psychology, 28*(1), 23–39.

Liu, X., Yi, X., & Wan, L.C. (2022). Friendly or competent? The effects of perception of robot appearance and service context on usage intention. *Annals of Tourism Research*, *92*, 103324.

Papies, D. (2017). The ugly side of customer management – Consumer reactions to firm-initiated contract terminations. *International Journal of Research in Marketing, 34*, 829-850.

Leung, E., Lenoir, A.I., Puntoni, S., & van Osselaer, S. (2023). Consumer preference for formal address and informal address from warm brands and competent brands. *Journal of Consumer Psychology*, *33*(3), 546-560.

Leung, F.F., Kim, S., & Tse, C.H. (2020). Highlighting effort versus talent in service employee performance: customer attributions and responses. *Journal of Marketing, 84*, 106-121.

Lepthien, Anke & Papies, Dominik & Clement, Michel & Melnyk, Valentyna. (2017). The ugly side of customer management – Consumer reactions to firm-initiated contract terminations. *International Journal of Research in Marketing*, *34*(4), 829-850.

Li, Y., Liu, B., Zhang, R., & Huan, T.T. (2020). News information and tour guide occupational stigma: Insights from the stereotype content model. *Tourism Management Perspectives, 35*, 100711.

Maher, A.A., & Carter, L.L. (2011). The affective and cognitive components of country image : Perceptions of American products in Kuwait. *International Marketing Review, 28*, 559-580.

Malika, M., & Maheswaran, D. (2023). Busy or poor: How time or money scarcity cues differentially impact purchase decisions regarding service firms. *Journal of the Academy of Marketing Science*, 1-18.

Philipp-Muller, A., Costello, J.C., & Reczek, R.W. (2023). Get your science out of here: when does invoking science in the marketing of consumer products backfire? *Journal of Consumer Research, 49*(5), 721-740.

Pizzi, G., Vannucci, V., Mazzoli, V., & Donvito, R. (2023). I, chatbot! the impact of anthropomorphism and gaze direction on willingness to disclose personal information and behavioral intentions. *Psychology & Marketing*, *40*(7), 1372-1387.

Rojas-Méndez, J. I., & Davies, G. (2023). Promoting country image and tourism in new or underdeveloped markets. *Journal of Travel Research*, Ahead-of-print: https://doi.org/10.1177/00472875231164967

Scott, M. L., Bone, S. A., Christensen, G. L., Lederer, A., Mende, M., Christensen, B. G., & Cozac, M. (2023). Revealing and mitigating racial bias and discrimination in financial services. *Journal of Marketing Research*, Ahead-of-print: https://doi.org/10.1177/00222437231176470

Scott, M. L., Mende, M., & Bolton, L. E. (2013). Judging the book by its cover? how consumers decode conspicuous consumption cues in buyer–seller relationships. *Journal of Marketing Research*, *50*(3), 334-347.

Singh, S.K., Marinova, D., Singh, J., & Evans, K.R. (2018). Customer query handling in sales interactions. *Journal of the Academy of Marketing Science, 46*, 837-856.

Wang, D., Oppewal, H., & Thomas, D.M. (2014). Exploring attitudes and affiliation intentions toward consumers who engage in socially shared superstitious behaviors: A Study of Students in the East and the West. *Psychology & Marketing, 31*, 203-213.

Wang, Z., Mao, H., Li, Y. J., & Liu, F. (2017). Smile big or not? Effects of smile intensity on perceptions of warmth and competence. *Journal of Consumer Research*, *43*(5), 787-805.

Wu, J., Chen, J., & Dou, W. (2017). The Internet of Things and interaction style: the effect of smart interaction on brand attachment. *Journal of Marketing Management, 33*, 61 - 75.

Xu, X., & Liu, J. (2022). Artificial intelligence humor in service recovery. *Annals of Tourism Research*, *95*, 103439.

Yoganathan, V., Osburg, V.S., & Kunz, W.H., & Toporowski, W. (2021): Check-in at the Robo-desk: Effects of automated social presence on social cognition and service implications. *Tourism Management*, *85*, 1-16,

Zhang, K., Li, S., & Ng, S. (2021). Sizes are gendered: the effect of size cues in brand names on brand stereotyping. *Journal of Consumer Research*, *49*(2), 252–267.

Zhang, L., Hanks, L., & Line, N.D. (2018). The joint effect of power, relationship type, and corporate social responsibility type on customers’ intent to donate. *Journal of Hospitality & Tourism Research, 43*, 374-394.

Zheng, X., Xu, J., & Shen, H. (2021). To be respected or liked: The influence of social comparisons on consumer preference for competence- versus warmth-oriented products. *International Journal of Research in Marketing*, *39*(1), 170-189.

Zhou, X., Kim, S., & Wang, L. (2019). Money helps when money feels: money anthropomorphism increases charitable giving. *Journal of Consumer Research, 45*, 953-972.
